# Structure and Surface Passivation of Ultrathin Cesium Lead Halide Nanoplatelets Revealed by Multilayer Diffraction

**DOI:** 10.1021/acsnano.1c08636

**Published:** 2021-11-29

**Authors:** Stefano Toso, Dmitry Baranov, Cinzia Giannini, Liberato Manna

**Affiliations:** † Nanochemistry Department, Istituto Italiano di Tecnologia, Via Morego 30, 16163 Genova, Italy; ‡ International Doctoral Program in Science, Università Cattolica del Sacro Cuore, 25121 Brescia, Italy; § Istituto di Cristallografia - Consiglio Nazionale delle Ricerche (IC−CNR), Via Amendola 122/O, I-70126 Bari, Italy

**Keywords:** multilayer diffraction, nanoplatelet, lead halide perovskite, Ruddlesden−Popper, X-ray, surface, structure

## Abstract

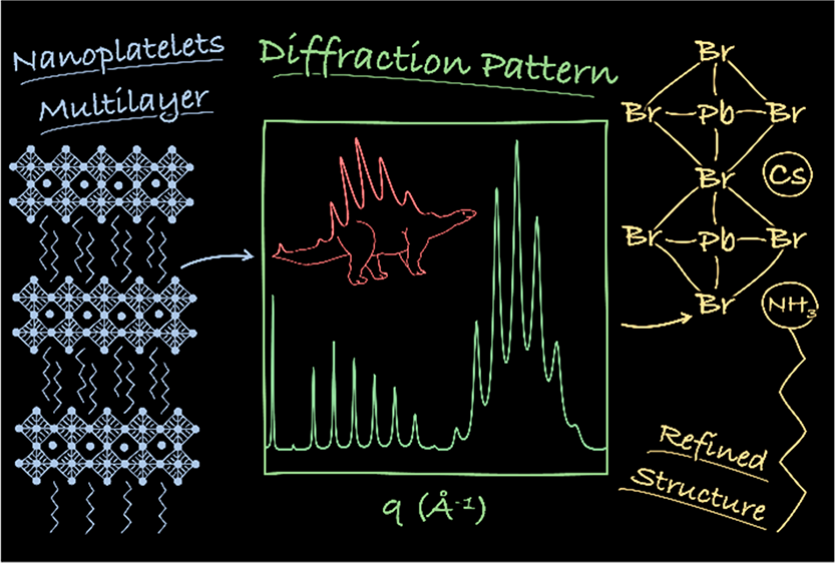

The research on two-dimensional colloidal semiconductors has received a boost from the emergence of ultrathin lead halide perovskite nanoplatelets. While the optical properties of these materials have been widely investigated, their accurate structural and compositional characterization is still challenging. Here, we exploited the natural tendency of the platelets to stack into highly ordered films, which can be treated as single crystals made of alternating layers of organic ligands and inorganic nanoplatelets, to investigate their structure by multilayer diffraction. Using X-ray diffraction alone, this method allowed us to reveal the structure of ∼12 Å thick Cs–Pb–Br perovskite and ∼25 Å thick Cs–Pb–I–Cl Ruddlesden–Popper nanoplatelets by precisely measuring their thickness, stoichiometry, surface passivation type and coverage, as well as deviations from the crystal structures of the corresponding bulk materials. It is noteworthy that a single, readily available experimental technique, coupled with proper modeling, provides access to such detailed structural and compositional information.

Colloidal semiconductor nanoplatelets are materials characterized by large exciton binding energy and oscillator strength, sharp spectral features,^1−4^ and short photoluminescence lifetimes,^2^ all properties that make them appealing in devices such as light-emitting diodes and lasers.^2,5−7^ The extreme thinness, down to just a few atoms, is both the source of their unique optical properties and a major challenge for their structural characterization. Several studies have demonstrated that nanoplatelets can be much different from an ideal “slice” of the corresponding bulk material: they are structurally less constrained than the bulk and can readily relax through structural distortions and altered lattice constants.^8^ Two examples are the rock-salt cubic PbS, which becomes orthorhombic when shaped into thin platelets,^9^ and the CdSe zincblende nanoplatelets, which are better described by a tetragonal structure rather than the cubic structure of the bulk.^10^ Furthermore, due to their high surface-to-volume ratio, the surface layers become a relevant fraction of the whole nanoplatelet, to the point that the type of surface termination affects the overall stoichiometry of the platelets and their physical properties.^2,11^

The emergence of colloidal lead halide perovskites considerably expanded interest in nanoplatelets in the past five years. Lead halide perovskite nanoplatelets can be synthesized with excellent control over their thickness from six down to one single [PbX_6_]^4–^ octahedron, providing access to a level of quantum confinement that would be challenging to achieve in isotropic nanocrystals.^1,2,8,12^ However, significantly less is known about these particles with respect to their cuboidal nanocrystal counterparts. Because of the intrinsic structural softness of halide perovskites and the symmetry breaking due to their finite thickness,^13−16^ such platelets may undergo a structural reorganization if compared to the bulk, which is challenging to capture in its fine details. Moreover, the extreme thinness of platelets makes their surface a considerable fraction of their entire volume, therefore requiring a careful investigation of their termination layers. Finally, films of lead halide perovskite nanoplatelets are actively explored for use in light-emitting devices. In such films, the platelet orientation, the interplatelet distance, and the stacking disorder affect the electronic coupling between nanoplatelets, their dielectric screening, and the orientation of their transition dipole moments. Therefore, such parameters can be relevant for tuning the optical characteristics of the device.^17−19^

To retrieve all this information, a combination of several techniques is typically required. For example, high-resolution transmission electron microscopy (HRTEM) is commonly used to investigate the structure of nanoplatelets.^20^ These, however, often lie flat with respect to the electron beam, and TEM is blind to their more elusive thin dimension unless in perfectly vertical stacks, a condition that is rarely met for thin and laterally extended platelets.^21^ Furthermore, curling and bending can prevent atomic resolution in ultrathin nanoplatelets, and the electron beam can cause severe damage.^8,10,22^ X-ray powder diffraction is a less invasive and more versatile and sensitive technique than TEM, but diffraction signals from nanoplatelets are smeared due to their limited periodicity along the thin direction. Thus, Bragg diffraction and its derived methods such as Rietveld refinement are poorly suited to characterize nanoplatelets, which are better studied with Total Scattering methods.^8,10^ However, these methods rely on high-quality data, usually from synchrotrons,^23^ and require an *a priori* 3D atomistic model of the entire nanoparticle to start with. Moreover, Total Scattering methods are usually applied on samples composed of randomly oriented crystallites: their extension to films of oriented nanoparticles requires the introduction of corrections for the preferred orientation, which would add further complexity to the method.

In this work, we investigate the structure of colloidal cesium lead halide nanoplatelets along their thinnest dimension through the application of Multilayer Diffraction to highly ordered nanoplatelet films. This technique has first emerged as a powerful and nondestructive analytical method following advances in the preparation of atomically precise multilayer structures.^24,25^ It is nowadays well-established for materials grown by physical methods,^24,26−32^ to the point that several user-written programs have been developed to apply the theory of Multilayer Diffraction to a variety of materials, such as metals, oxides, or organic multilayers.^28,33−36^ Our groups recently expanded the domain of application of Multilayer Diffraction to superlattices of colloidal semiconductor nanocrystals.^37,38^ Briefly, these materials can be considered as multilayers where high-density inorganic domains (*i.e.*, the nanocrystals) alternate with low-density organic layers (*i.e.*, the ligands), whose scattering power can be neglected or approximated. Consequently, their diffractograms can be simulated by first computing the diffraction profile of the individual nanoparticles and then including the effects of the multilayer interference phenomena arising from the precise particle stacking. This allows us to extract the sample structural parameters by performing a least-squares refinement of the experimental diffractogram. In the present work, we further developed this approach by introducing a fully atomistic description of the nanoplatelet structure, in contrast to the previous approximation of the nanocrystal as a stacking of scattering planes with no chemical identity.^38^ This enabled us to refine the atomic structure of the constituent nanoplatelets, greatly increasing the potential of the method.

The single- and mixed-halide Cs–Pb–X (X = Br or I–Cl) nanoplatelets analyzed in this work were prepared by colloidal synthesis and self-assembled into thin films by slow solvent evaporation. Such a self-assembly process was exploited to create an extended periodicity along the thin direction of the nanoplatelets, producing a peculiar pattern of periodic peaks when the film is subject to a θ:2θ diffraction experiment. By applying the knowledge derived from our previous work on lead halide nanocrystal superlattices,^37,38^ we developed an algorithm that enables the refinement of the nanoplatelets structure by means of a full-profile multiparametric fitting of their diffractogram. Despite relying only on a widely available lab-grade diffractometer, the amount of structural and compositional information that can be retrieved is noteworthy. When the method was applied to Cs–Pb–Br nanoplatelets, it demonstrated that they were two-monolayer (*i.e.*, two [PbBr_6_]^4–^ octahedra) thick, stacked at about ∼34 Å from each other with a standard deviation of only ∼0.5 Å. Furthermore, the method enabled us to discern the surface termination of the platelets, which consisted of a partially defective plane of Br^–^ ions and R-NH_3_^+^ ligands, and to demonstrate that oleic acid, although present during the synthesis, played no role in their surface passivation. We also measured the anisotropic structural expansion of nanoplatelets with respect to bulk CsPbBr_3_, confirming a behavior reported for cesium lead halide nanostructures in the past.^8,38,39^

While a relatively simple structure and a well-documented relationship between thickness and optical properties ease the identification of the number of lead halide octahedra layers in the case of Cs–Pb–Br nanoplatelets, the presented method enables the investigation of more elusive materials as well. Here, we report the example of Cs_2_PbI_2_Cl_2_ Ruddlesden–Popper nanoplatelets, for which the number of layers cannot be determined from optical spectra. Nonetheless, for this system the Multilayer Diffraction approach enabled us to accurately measure a thickness of three [PbI_2_Cl_4_]^4–^ octahedra, capture the anisotropic expansion of the structure along its thin dimension, and determine the nature of the surface termination layer, which was found to be composed of I^–^ and R-NH_3_^+^ ions. Contrary to our initial expectations, we also found that the Cs^+^ and I^–^ ions form a common atomic plane in the nanoplatelets, as opposed to being located in separate planes as in bulk Cs_2_PbI_2_Cl_2_, providing yet another example of structural differences between a nanoparticle and the corresponding bulk material.

## Results and Discussion

### Sample Preparation, Data Processing, and Model Outline

**Figure 1:**
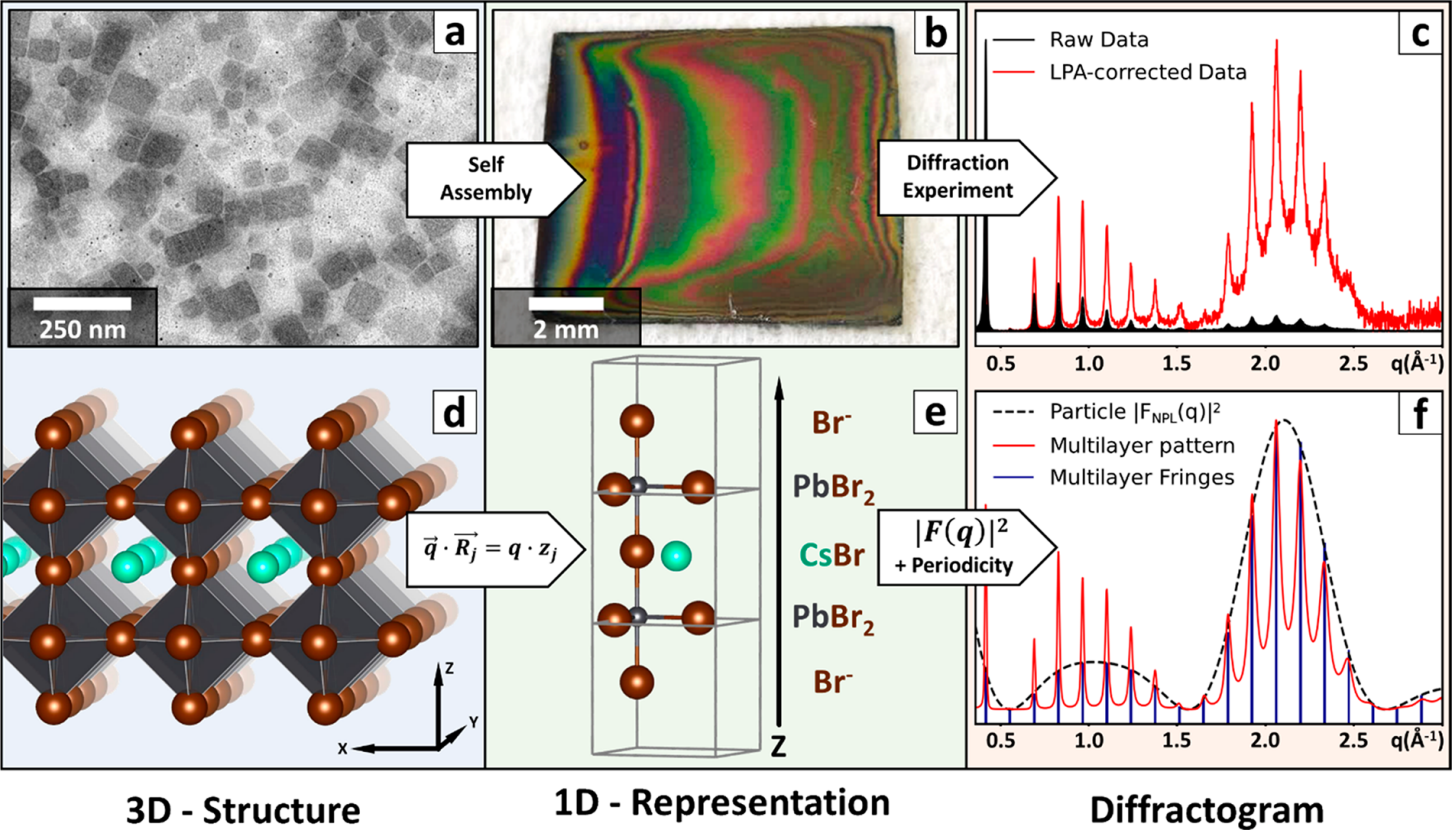
Experimental and simulated multilayer diffraction patterns. The slow solvent evaporation drives the self-assembly of (a) colloidal nanoplatelets of uniform thickness into (b) highly oriented iridescent thin films on top of a silicon wafer (1 cm × 1 cm). In such films, the electron-dense platelets and the comparatively electron-light ligands alternate along the vertical direction. These are multilayered systems that modulate the intensity of the diffracted X-rays yielding patterns into characteristic periodic sharp fringes. Such experimental patterns (c, black solid line “Raw Data”) can be conveniently exploited to refine the structure of the nanoplatelets by comparing them with a simulation once the Lorentz Polarization Absorption correction has been applied (c, red solid line “LPA-corrected data”). Indeed, the ordered stacking into a multilayer allows the reduction of the 3D structure of randomly oriented nanoplatelets (d) to a 1D representation of the film along its *z*-axis (e). Such representation is used to compute the nanoplatelet structure factor *F*_NPL_(*q*) through eq 2 and then to convolute its square modulus |*F*_NPL_(*q*)|^2^ (f, black dashed line) with the fringes arising from the nanometer-scale periodicity of the multilayer (f, vertical blue lines). This produces a simulation of the whole multilayer diffractogram (f, red solid line) that can be matched with the experimental data to refine the input 1D representation of the nanoplatelet structure.

In this work, we prepared Cs–Pb–Br nanoplatelets with a lateral dimension of a few dozens of nanometers (Figure 1a) *via* a previously published procedure.^40^ The films of oriented nanoplatelets for the Multilayer Diffraction analysis were prepared by slowly drying a suspension of nanoplatelets in hexane on top of a tilted silicon wafer. Upon the successful film deposition, a region of the substrate became covered by a smooth and iridescent film and was selected for the diffraction experiment. As a matter of fact, a brilliant and homogeneous color over mm^2^ areas, originated by thin-film light interference, attests to the expected constant thickness and limited roughness of the film (Figure 1b).^41^ The samples were probed in θ:2θ out-of-plane diffraction experiments, producing signals in the form of equally spaced fringes whose intensity and sharpness decreased at higher angles. Such diffraction fringes appear when the structural perfection of the multilayer reaches the angstrom level,^38^ far beyond what can be probed optically, and encode the structure of the film along its vertical direction. This includes the overall periodicity of the multilayer and its stacking disorder, as well as the vertical coordinates and the electron densities of all the atomic planes in the sample.

To extract this information, we refined a starting model of the multilayer by minimizing the differences between the experimental data and a simulated diffractogram, similarly to what is done in a Rietveld refinement. First, the data must be prepared for the analysis by subtracting the instrumental background and any residual reflection from misaligned nanoplatelets or from the substrate. In addition, the Lorentz Polarization Absorption correction must be applied to compensate for the instrumental contributions to the measured intensity (LPA, Figure 1c).^28^ This step is needed to make the experimental pattern comparable with the simulation. Indeed, the so-processed data are an experimental measurement of |*F*_ML_(*q*)|^2^, that is the square modulus of the multilayer structure factor *F*_ML_(*q*) describing the amplitude and phase of X-rays diffracted by the multilayer. Our analysis relies on the fact that *F*_ML_(*q*) can be simulated starting from a structural representation of the multilayer.

To achieve such a description, the multilayer can be broken down into two parts: the high-density inorganic nanoplatelets and the low-density interparticle spacings composed of organic ligands. Due to their low density, the interparticle spacings minimally contribute to the signal intensities across the experimentally studied q-range, and for the purpose of the analysis they can be approximated as amorphous carbon layers with fixed density. The most important part of the multilayer are the nanoplatelets, whose structure factor *F*_NPL_(*q*) is the main component of *F*_ML_(*q*). In this work, we describe *F*_NPL_(*q*) in its most essential form, that is, the sum in phase of the radiation scattered by each individual atom *j*(42)
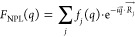
1where *f*_*j*_ is the element-specific atomic form factor, *q⃗* is the scattering vector, and *R⃗*_*j*_ = *x̂*·*x*_*j*_ + *ŷ*·*y*_*j*_ + *ẑ*·*z*_*j*_ is the position vector of the *j*th atom inside the nanoplatelet. Computing the structure factor in this way would be demanding for platelets containing dozens of thousands of atoms; however, developing eq 1 one step further immediately highlights the huge advantage of dealing with ordered multilayers instead of randomly oriented nanoplatelets. During a θ:2θ out-of-plane diffraction experiment, the scattering vector *q⃗* is perpendicular to the sample surface plane *xy*; therefore, *q⃗*·*R⃗*_*j*_ = *q*·*z*_*j*_:
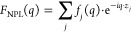
2As small as it might seem, this change has a major impact on the data treatment. Instead of considering a complex 3D model (Figure 1d), we can describe the structure entirely by the handful of atoms needed to capture the composition of each atomic plane along the *z* direction. For example, two [PbBr_6_]^4–^ octahedra thick Cs–Pb–Br nanoplatelets are described by just 10 atoms divided in five layers: Br–PbBr_2_–CsBr–PbBr_2_–Br (Figure 1e). Based on this representation, our algorithm computes *F*_NPL_(*q*) and then exploits it to calculate *F*_ML_(*q*) by introducing the effects of the multilayer periodicity and stacking disorder. Finally, *F*_ML_(*q*) is turned into |*F*_ML_(*q*)|^2^, producing a simulation that can be matched with the experimental data (Figure 1f). Additional details on the sample preparation, the data analysis, and the diffractogram simulation can be found in the Methods and in the Supporting Information (SI), sections S1–S3.

### Structural Refinement of Cs–Pb–Br Nanoplatelets

**Figure 2:**
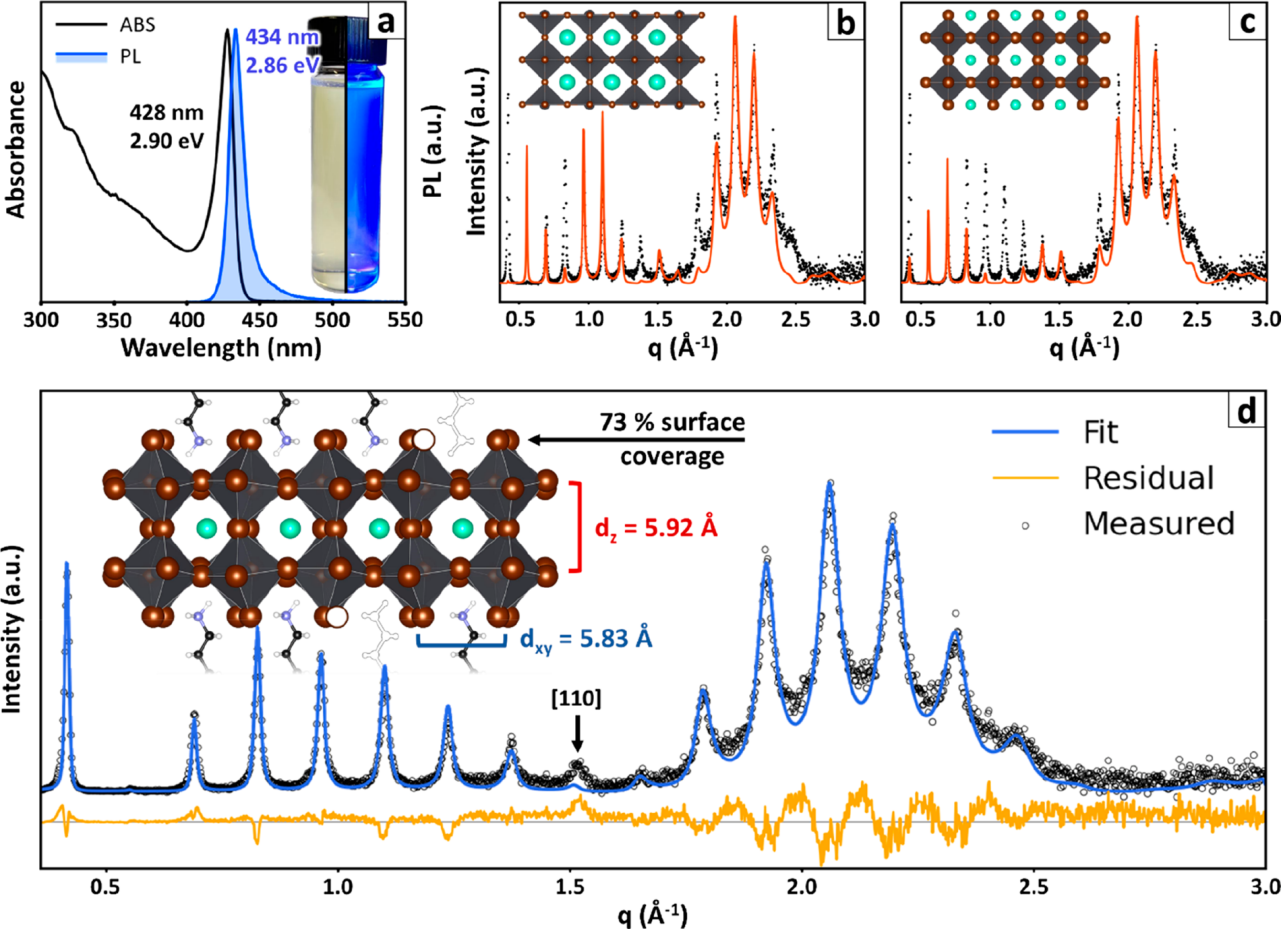
Structural refinement of Cs–Pb–Br nanoplatelets. (a) Absorption (ABS) and photoluminescence (PL) spectra of two [PbBr_6_]^4–^ octahedra thick Cs–Pb–Br nanoplatelets. Inset: a photograph of a colloidal suspension of nanoplatelets under ambient illumination (left) and UV light (right), the latter showing the characteristic bright blue emission. Preliminary simulations testing the hypotheses of (b) a PbBr_2_ surface termination and (c) a CsBr surface termination. A visual examination reveals that these surface terminations do not capture accurately the intensity of the peaks in the experimental patterns. (d) Best-fit of the diffraction pattern obtained by refining the structural parameters of the Br^–^/R-NH_3_^+^ termination model, including the occupancies, and the vertical coordinates of atoms in the platelet structure. The residual signal at *q* ∼ 15 Å^–1^ is not a multilayer fringe, but a peak from a fraction of misaligned platelets ({110} in the pseudocubic notation for CsPbBr_3_). The inset illustrates the nanoplatelet structure in scale, highlighting its expansion along the vertical direction, the partially occupied surface layers and the tilted [PbBr_6_]^4–^ octahedra. Color legend for atoms: Cs^+^ = light-blue; Br^–^ = brown; Pb^2+^ = dark gray (octahedra); *N* = violet; C = black; H = white.

The model we outlined allows us to refine the structure of Cs–Pb–Br nanoplatelets *via* the optimization of a consistent starting model, which is a 1D representation of the nanoplatelets where the number of atomic planes, their stacking sequence, and the elements they contain is imposed *a priori*. For quantum-confined platelets, the thickness can be estimated through the absorption spectrum: in our case, it matched with reports for ∼12 Å thick nanoplatelets (excitonic absorption peak at 428 nm, Figure 2a).^12,40,43^ Taking the CsPbBr_3_ crystal structure as a reference, where the Pb–Br bonds are about ∼3 Å long, the nanoplatelets must be composed of five alternating CsBr and PbBr_2_ layers. To complete the nanoplatelet model, we had to provide a description for the surface as well. Indeed, as a consequence of the extreme thinness the surface layers are a significant fraction of the entire nanoplatelet, as they constitute two out of five atomic layers along the thin direction. Such a high contribution is expected to leave a clear mark on the diffraction pattern, therefore giving insight into the nature of the surface passivation layer. To elucidate this aspect, we considered three surface models that have been previously proposed for CsPbBr_3_ nanocrystals: PbBr_2_-termination, CsBr-termination, and Br^–^/R-NH_3_^+^ termination (Figure 2b–d, insets).^44−49^ A preliminary comparison between calculated and experimental patterns, performed by refining only a few selected parameters (Pb–Pb distance, interparticle spacing, stacking disorder), easily discarded the PbBr_2_- and CsBr-terminations (Figure 2b,c), while the Br^–^/R-NH_3_^+^ termination produced a good match with the experimental data (SI, section S4).

The match between the measured and calculated diffractograms could be further improved by refining the fractional occupancies of the atoms in each plane, which is described in the model by multiplying each atomic form factor *f*_*j*_ in eq 2 by a number in between 0 (zero occupancy) and 1 (full occupancy). The resulting picture of the nanoplatelets structure is hereby presented (Figure 2d, see the SI, section S5, for further details on the analysis). The Cs–Pb–Br nanoplatelets were confirmed to be composed of two [PbBr_6_]^4–^ octahedra layers, for an overall thickness of 11.85 ± 0.01 Å, and were separated by a layer of organic ligands 34.00 ± 0.01 Å thick with a stacking disorder parameter of 0.51 ± 0.01 Å (*i.e.*, the standard deviation of the interparticle distance). This corresponds to an overall multilayer periodicity of 45.89 ± 0.01 Å. The most electron-dense atomic layers, namely the PbBr_2_ ones, were assumed to be fully occupied to serve as a reference for refining the relative occupancies of all the other layers. With respect to this reference, the CsBr layer in the middle of the platelet was found to be slightly to nondefective (occupancy = 95 ± 1%), while the surface Br^–^/R-NH_3_^+^ layer was significantly defective (occupancy = 73 ± 2%). The nanoplatelet structure was found to be expanded along the thin direction: the Pb–Pb distance is 5.924 ± 0.004 Å, longer than that measured for the horizontal direction by 1.56% (5.833 Å, from the residual signal of nonstacked platelets, see the SI, section, S2). Furthermore, both Pb–Pb distances are longer than those found in bulk CsPbBr_3_ (vertical = +0.75% [010] bulk, horizontal = +0.22% ⟨101⟩ bulk, *Pnma* setting, ICSD-97851).^50^ This finding is in line with the known tendency of lead halides to relax their structure anisotropically at the nanoscale^8,38^ and provides insight about the lattice symmetry.

Lead halide perovskites can adopt a cubic, tetragonal, or orthorhombic structure depending on whether and how the [PbX_6_]^4–^ octahedra are tilted.^51,52^ While the debate on bulk and large nanocrystals of CsPbBr_3_ points in favor of the orthorhombic polymorph under normal temperature and pressure, this aspect is still not entirely clarified for small nanocrystals and thin platelets.^53−57^ The different Pb–Pb distances along the horizontal and vertical directions easily exclude the cubic symmetry: we tried to discern between the two options left based on the atoms in the PbBr_2_ planes being coplanar (tetragonal) or misaligned (orthorhombic) due to the tilting of octahedra (see SI, Section S5, for details). To do so, we allowed the *z*-coordinate of the Br^–^ ions in the PbBr_2_ planes to relax, finding that |*z*_Pb_–*z*_Br_| = 0.241 ± 0.054 Å. This is compatible within error with the |*z*_Pb_–*z*_Br_| = 0.294 Å found in bulk CsPbBr_3_,^50^ suggesting that the two-monolayer Cs–Pb–Br nanoplatelets adopt an orthorhombic structure, in line with a previous report on six-octahedra-thick nanoplatelets.^8^ See the SI, Section S5, for further details on the analysis of Cs–Pb–Br nanoplatelets.

### On the Surface Passivation of Cs–Pb–Br Nanoplatelets

X-ray diffraction is not often exploited to study the surface of nanocrystals due to the predominant contribution of the volume to the overall diffracted intensity.^58^ However, assembling the platelets into a multilayer effectively turned their surface into a periodic component of a larger crystal, therefore enabling its investigation. The diffraction methods are sensitive to the electron density of the sample, which depends on its chemical composition. Thus, the nanoplatelet surface experimentally appears as a layer of atoms with a lower electron density than the core, to which we must give an interpretation in terms of chemical composition. This is done by assuming a starting model passivated by a layer of Br^–^ and R-NH_3_^+^ ions, in line with prior findings and with our preliminary simulation (Figure S7a), and then refining the occupancy of surface ions by matching the electron density of the model with the one measured experimentally. For example, the 73% occupation found for the Br^–^/R-NH_3_^+^ surface layers indicates that they have an electron count of ∼31.4 e^–^/formula unit (= 0.73·[e^–^_Br_ + e^–^_N_]) if compared to the PbBr_2_ layers we took as a reference (152 e^–^/formula unit). The raw data is the electron count, while the vacant layer of Br^–^/R-NH_3_^+^ ions is the way our model accounts for it. In principle, many other surface terminations are compatible with the same electron count and would be indistinguishable by relying only on the diffraction data from our experiments. Therefore, we had to validate this interpretation by independent methods.

The first hint toward a Br^–^/R-NH_3_^+^ termination is provided by the comparison with 2D-layered perovskites. Those are bulk materials where layers of A^+^ cations of the perovskite (*i.e.*, Cs^+^ for CsPbBr_3_) are replaced with a long-chain ammonium cation, that has the effect of dissecting the perovskite structure into inorganic slices separated by the organic component.^59,60^ At the interface between the inorganic and the organic domains of the crystal, the polar heads of the ammonium cations replace the A^+^ cations. The same happens in our nanoplatelets films, where thin inorganic nanoplatelets are separated by organic molecules: it is fair to assume that the Cs^+^ ions on the surface are replaced by the polar heads of oleylammonium ions. Based on this assumption we can calculate the theoretical elemental ratio in the sample. For vacancy-free two octahedra-thick platelets the calculated ratio is Cs/Pb/Br = 1:2:7, or otherwise 0.95:2:6.41 if the occupancies from the fit are included. By SEM-EDS we measured Cs/Pb/Br = 0.93:2:6.43, while by XPS we found Cs: Pb/Br = 1:2:6.1 (see the SI, section S6). In both cases, the Pb/Cs ratio is consistent with the model, demonstrating that no Cs^+^ is found on the surface of the platelets. The amount of bromine measured by SEM-EDS is also compatible with the expectations, while it is slightly lower by XPS. In the latter case, the lower Br content is rationalized as either due to a beam-induced Br desorption or to the segregation of defective platelets on the film surface (that is the spatial region to which XPS is most sensitive) during the self-assembly process.

Another open question is whether oleic acid plays a role in surface passivation of the nanoplatelets.^45^ Both oleylamine and oleic acid are used in the synthesis, and while an oleylammonium bromide termination easily accounts for the role of oleylamine, the presence of oleic acid cannot be easily excluded. We addressed this question by characterizing self-assembled nanoplatelets that had been synthesized with either a longer carboxylic acid (erucic acid with C_22_ as compared to C_18_ in oleic acid) or a shorter amine (octylamine with C_8_ as compared to C_18_ in oleylamine), taking advantage of the sensitivity of Multilayer Diffraction to the interparticle spacing. The introduction of erucic acid produced no appreciable change in the diffractogram. Instead, in the octylamine-containing sample the interparticle distance dropped from 34.0 to15.2 Å and the stacking disorder parameter decreased from 0.50 to 0.24 Å. These findings confirmed the hypothesis that oleic acid plays no significant role in the passivation of the nanoplatelet surface, and are consistent with a prior NMR study by de Roo *et al.* demonstrating that oleic acid is not interacting with the surface of CsPbBr_3_ nanocrystals in liquid dispersion.^45^ See the SI, Section S6 for data and details regarding the investigation of the surface passivation.

### Identification and Refinement of the Cs–Pb–I–Cl Nanoplatelet Structure

We then turned our attention to mixed-halide I–Cl nanoplatelets, a system that offers an increased structural complexity due to the presence of two different anions which can, in principle, share the same positions within the structures of cesium lead halides. This combination of halides is appealing for a diffraction analysis due to the large difference in the electron densities between Cl^–^ and I^–^, which makes it easier to tell them apart, and because the different ionic radii favors the segregation of halides, inducing the formation of the Cs_2_PbI_2_Cl_2_ Ruddlesden–Popper phase.^63^ Only a few reports were published on nanocrystals of this compound, and a detailed characterization of their structure is lacking to date.^61,62,64,65^

**Figure 3:**
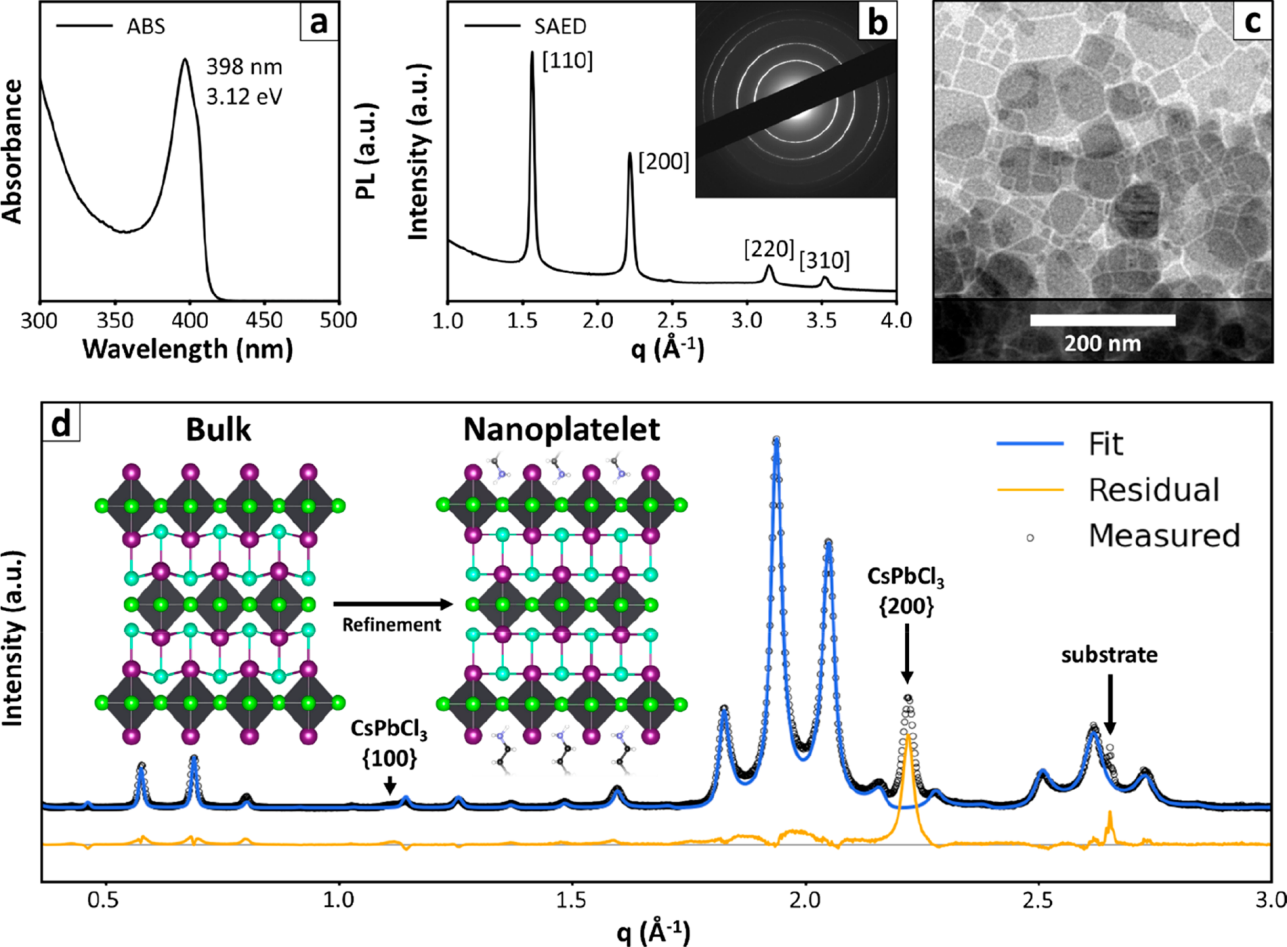
Cs–Pb–I–Cl Ruddlesden–Popper nanoplatelets. (a) Absorption spectrum of Cs–Pb–I–Cl nanoplatelets. The spectral position of the excitonic peak matches that reported in the past for nanocrystals of the same material (400 nm/3.10 eV),^61,62^ and is only weakly shifted from that of bulk Cs_2_PbI_2_Cl_2_ (408 nm/3.04 eV).^63^ (b,c) TEM images and SAED diffraction pattern of Cs_2_PbI_2_Cl_2_. The SAED pattern provides information about the structure of the platelets along the horizontal direction and is compatible with what is expected for oriented Cs_2_PbI_2_Cl_2_ crystals. (d) Multilayer Diffraction fit of the Cs–Pb–I–Cl nanoplatelets pattern. The starting model for the fit was a slice of the published bulk structure (ICSD-6337),^63^ I^–^/R-NH_3_^+^ terminated, with a thickness of three [PbI_2_Cl_4_]^4–^ octahedra. In the refined structure, the Cs^+^ and I^–^ ions are found to converge into one common layer of ions as the platelet expands slightly along the vertical direction (Pb–Pb distance +1.4%). The residual nonfitted signals correspond to the {100} and {200} Bragg peaks of the CsPbCl_3_ impurity and to some signals from the substrate. Color legend for atoms: Cs^+^ = light-blue; Cl^–^ = green; I^–^ = purple; Pb^2+^ = dark gray (octahedra); N = violet; C = black; H = white.

The Cs–Pb–I–Cl nanoplatelets were prepared by a modification of the protocol used for Cs–Pb–Br nanoplatelets. The modifications consisted in using a doubled amount of Cs-oleate and in injecting a 1:1 mixture of benzoyl chloride and iodide at 50 °C. Upon injection, the solution immediately turned red, followed by a color transition to light red-orange after a minute of growth, and to pale yellow upon the addition of ethyl acetate during the nanoplatelet isolation. The synthesized particles were analyzed by absorption spectroscopy and TEM (Figure 3). The absorption spectrum features a strong excitonic peak at 3.12 eV, compatible with that reported in the past for Cs_2_PbI_2_Cl_2_ nanoplatelets.^61,62^ It is worth noting that in Cs_2_PbI_2_Cl_2_ the exciton absorption position is weakly dependent on the thickness of nanocrystals, as the exciton peak in the bulk is red-shifted by only 0.08 eV (being at 3.04 eV). This is because the [PbX_6_]^4–^ octahedra are disconnected along the *c* crystallographic axis, and behave as individually confined systems regardless of the thickness of the crystal.^61^ Therefore, the thickness of the platelets had to be inferred directly from the diffraction pattern. The sample was self-assembled into a multilayer film and analyzed through X-ray diffraction, producing a pattern that appeared very different from the case of bromine-based platelets: the most intense group of fringes was found at a different position in the *q-*scale (∼2 Å^–1^), and was less broad, suggesting thicker platelets than in the previous case. Furthermore, we identified two diffraction features that did not belong to the general periodicity of the multilayer, located at *q* = ∼1.12 Å^–1^ and *q* = ∼2.22 Å^–1^. The {100} and {200} Bragg peaks of pseudocubic CsPbCl_3_^66,67^ provided a potential match for these diffraction features, possibly indicating an impurity of this compound in the sample. Since their overlap with the Cs–Pb–I–Cl diffraction features is minimal, we decided not to subtract them to minimize unnecessary data processing.

In order to find a suitable starting model for the refinement of Cs–Pb–I–Cl nanoplatelets we tested several possible structures obtained by slicing the reported bulk structure, until we found a good match withthree [PbX_6_]^4–^ octahedra thick, I-/R-NH_3_^+^ terminated Ruddlesden–Popper nanoplatelets (see the SI, Section S7). Starting from this preliminary model, we refined the atomic coordinates and occupancies of the atoms on the surface. The platelets were found to be 25.11 ± 0.03 Å thick, and were separated by an interparticle layer of 30.25 ± 0.08 Å with a stacking disorder parameter of 0.41 ± 0.07 Å. This corresponds to an overall multilayer periodicity of 55.36 ± 0.08 Å. Like the Cs–Pb–Br platelets, the crystal structure was slightly expanded along the thickness of the platelets (Pb–Pb vertical distance = 9.58 Å *vs* 9.44 Å published,^63^ + 1.46%). The surface coverage was found to be 90 ± 6%, higher than that measured for pure bromide nanoplatelets.

Thanks to the excellent quality of the diffractogram, we could also refine the coordinates of the atomic planes within the platelets. Interestingly, the Cs^+^ and I^–^ ions were found to be almost coplanar (|*z*_Cs_–*z*_I_| = 0.17 ± 0.15 Å), whereas in the structure reported for bulk Cs_2_PbI_2_Cl_2_ they are shifted by 0.68 Å, therefore forming strongly staggered planes. This is most likely an effect of the expansion along the thin direction, that stretches the structure forcing the alignment. To conclude, we remark that the fit produced excellent results even if the iodide and chloride atoms were assigned to strictly different crystallographic positions, without the need of considering mixed occupancies. This indicates that the two halides segregate completely within the nanoplatelets, a result that is expected based on the crystal structure of bulk Cs_2_PbI_2_Cl_2_, but that is not trivial for ultrathin nanoplatelets exposed to a reaction environment containing an excess of both Cl^–^ and I^–^ ions. The proposed structural model corresponds to a composition of Cs/Pb/I/Cl = 4:3:5.8:6. The experimental composition of the sample was found to be Cs/Pb/I/Cl = 4.8:4.3:5.8:9.2 by SEM-EDS and 7.0:5.6:5.8:12.2 by XPS (see the SI, Section S8). After subtracting the predicted ratio, the measured compositions leave Cs/Pb/Cl = 0.8:1.3:3.2 ratio (from SEM-EDS result) and 3:2.6:6.2 (from XPS result). Such residuals are consistent with a CsPbCl_3_ impurity in the sample, which was also indicated by XRD. The higher fraction of impurity detected by XPS is explained by a segregation of CsPbCl_3_ at the top of the nanoplatelet film, which, again, is the region that contributes the most to the XPS signal. It is indeed worth noting that any impurity situated in between the substrate and the multilayer would inevitably increase the measured stacking disorder of the multilayer, which is not the case for our samples.

## Conclusions

The self-assembled periodic stacks of cesium lead halide nanoplatelets can be considered the analogue of a hybrid organic–inorganic single crystal, at least along the probed *z*-direction. In such stacks, the nanoplatelets, their surface layers, and the organic ligands between them constitute the structural motif that is repeated by the periodicity of the multilayer. Such order is exclusive to the stacking direction, as the samples are polycrystalline in-plane. However, the in-plane mosaicity of the film does not impact our analysis, as the experiments we reported selectively probe the sample perpendicularly to the substrate.

In analogy with the refinement procedures adopted for conventional crystals, the multilayer structure can be refined through a multiparametric fit of its diffraction pattern. Given a starting structural model that qualitatively matches the experimental data, the refinement procedure allows to measure the multilayer periodicity, its structural disorder, and the interparticle distance. Furthermore, it gives access to the accurate determination of the nanoplatelets thickness, crystal structure, stoichiometry and even surface passivation. As a result, the method is able to provide insights into the surface composition, such as the determination of the surface coverage of nanoplatelets and the absence of oleic acid in their passivation layer. Furthermore, the analysis enables a detailed comparison between the structure of nanoplatelets and that of the corresponding bulk phases. This is the case of Cs_2_PbI_2_Cl_2_, where the Cs^+^ and I^–^ ions were found to be coplanar in nanoplatelets while they are misaligned in bulk crystals (see Figure 3d, inset).

While relevant *per se*, we predict that such insights will become even more significant for the rapidly growing field of self-assembled halide perovskite superstructures,^5,18,37,38,68−70^ where the methodology we have developed might complement or even outmatch more elaborate characterization tools within the limits imposed by probing a single direction. The Multilayer Diffraction approach we adopted provides indeed a large amount of detailed information, while relying solely on a simple and widely accessible θ:2θ out-of-plane diffraction experiment. Such experimental geometry is often used for *in situ* investigations and if applied to nanoplatelet multilayers would give the opportunity to monitor their structural response under external stimuli such as illumination, charge injection, temperature or pressure gradients, and exchange or intercalation of chemical species. To help further developments in this direction, we provided the simulated diffraction patterns for Cs–Pb–Br and Cs–Pb–I–Cl nanoplatelets of several different thicknesses (see the SI, section S9).

Finally, by performing a broad literature search we identified a large number of published diffraction patterns that could be analyzed with a Multilayer Diffraction approach. Those are recognizable by the equally spaced diffraction peaks whose intensity shows a collective trend (*e.g.*, decreasing in intensity toward higher *q* or, as in this work, organized in broad groups with maximum intensity at their center). Such diffraction patterns are often reported for other metal halides, both in the form of colloidal two-dimensional nanostructures or of bulk layered phases.^71−73^ However, we also found several other examples for metal oxides,^74−77^ metal hydroxides,^78,79^ natural layered silicates,^80^ and MXenes,^81,82^ both in the form of colloidal two-dimensional nanostructures and layered bulk crystals. For these materials, and for any others that exhibit a tendency to form stacks, the application of Multilayer Diffraction represents an opportunity to gain deeper insight into their compositional, structural, and surface-related properties.

## Methods

### Chemicals

Lead(II) acetate trihydrate (99.999%, Sigma-Aldrich), cesium acetate (99.99%, Sigma-Aldrich), benzoyl chloride (Bz-Cl, 99%, Sigma-Aldrich), benzoyl bromide (Bz-Br, 97%, Sigma-Aldrich), sodium iodide (99.5%, Sigma-Aldrich), decane (anhydrous, ≥99%, Sigma-Aldrich), oleylamine (technical grade, 70%, Sigma-Aldrich), oleic acid (technical grade, 90%, Sigma-Aldrich), octylamine (99%, Sigma-Aldrich), erucic acid (analytical standard, ≥99.0%, Sigma-Aldrich Supelco), hexane (puriss. p.a., ACS reagent, Sigma-Aldrich), ethyl acetate (puriss. p.a., ACS reagent, Sigma-Aldrich). All chemicals were used without further purification.

### Synthesis of Cs–Pb–X Nanoplatelets

All the nanoplatelets were prepared according to the same general procedure, which is here exemplified for the case of oleylammonium-capped Cs–Pb–Br nanoplatelets. The specific conditions applied for each sample discussed in this work are detailed in the SI, Table S1. First, a solution of ligands (120 μL of oleylamine and 160 μL of oleic acid) in 2 mL of decane was heated to 100 °C inside an 8 mL glass vial. Second, the metal carboxylates (60 μL of Pb-oleate and 20 μL of Cs-oleate) were preheated to the same temperature to achieve a good homogeneity and an adequate fluidity, and added to the solution by dipping and flushing the micropipette tips inside the liquid several times to ensure a quantitative release. Finally, the benzoyl halide (20 μL of Bz-Br) was injected in the solution at 100 °C, triggering the immediate nucleation of the nanoplatelets. After a growth time of 1 min, the reaction was quenched by immersing the vial in water at room temperature. The resulting colloidal suspension was destabilized by adding ethyl acetate until it turned cloudy (∼4 mL). The particles were recovered by centrifugation at 4000 rpm for 2 min and were resuspended in 1.2 mL of hexane. The so-obtained suspension was filtered with a 0.2 μm PTFE syringe filter and stored in a closed vial.

### Benzoyl Iodide Preparation

Benzoyl iodide (Bz-I) was prepared by mixing 1.4 mL of Bz-Cl and 3.0 g of sodium iodide at ∼75 °C overnight inside a N_2_-filled glovebox. The liquid was recovered with a syringe, filtered with a PTFE 0.2 μm filter, and collected inside a vial. After a few hours at room temperature a solid, likely a sodium halide, formed on the vial walls and was discarded by repeating the filtration process. The reaction product was an orange-red liquid, that could be stored inside a glovebox in a dark vial for more than one month without any appreciable change in its aspect or reactivity.

### Preparation of Metal Carboxylates

Lead oleate was prepared by mixing 379 mg (1 mmol) of lead acetate trihydrate with 1.5 mL of oleic acid at 100 °C for 3 h. The process was carried out under mild vacuum to ease the volatilization of acetic acid. Cesium oleate was prepared by mixing 192 mg (1 mmol) of cesium acetate with 1.0 mL of oleic acid at 100 °C for ∼1 h. As in the case above, the process was carried out under mild vacuum to ease the volatilization of acetic acid. Both processes yielded dense yellow liquids which readily solidified when cooled at room temperature. Metal oleates were preheated to 100 °C and stirred for a few minutes before taking any aliquot to ensure the compositional homogeneity of the precursor. Lead and cesium erucates were prepared in a similar way, simply replacing oleic acid with an equimolar amount of erucic acid. For Cs-erucate 1.07 g was used, while for Pb-erucate 1.61 g was used.

### Preparation of Nanoplatelet Films

The films for the Multilayer Diffraction experiments were prepared by diluting with hexane the nanoparticles solutions obtained as described above by a factor of 2–5. After the dilution, 50 μL of solution were carefully deposited on a 1 × 1 cm silicon wafer (Ted Pella, Inc., ⟨100⟩ orientation) placed inside a glass Petri dish (inner volume ∼25 cm^3^). Then, the Petri dish was closed with its glass lid, and a chink in between the edge and the lid was created by adding a small piece of folded aluminum foil. The solution was let drying completely until a homogeneous and iridescent film was formed: the process required about 2–5 min. If the film appeared opaque and not iridescent, the solution was further diluted, and the process repeated. If the film appeared too thin or invisible, a more concentrated solution was used instead. Figure S3 shows one successfully prepared film as seen from above. A 1 mm thick glass slide placed underneath the silicon to tilt it at an angle of about 10–15° helps in getting more homogeneous films on at least one region of the substrate. The thickness of the films in the iridescent areas was found to be around 0.5 μm (see SI, Figure S4).

### Diffraction Data Collection and Processing

The diffraction patterns of the nanoplatelet films were collected with a Panalytical Empyrean diffractometer in a parallel-beam configuration, equipped with a 1.8 kW Cu Kα ceramic X-ray tube operating at 45 kV, 1 mm wide incident and receiving slits, and a 40 mA PIXcel3D 2 × 2 two-dimensional detector. While the two-dimensional detector is not a strict requirement for the application of the Multilayer Diffraction method, it can help better visualizing the data and recognizing the signals coming from multilayer stacks of nanoplatelets from those produced by other sources (*e.g*. the substrate or misaligned particles). In fact, multilayer diffraction signals appear in the form of vertical straight stripes. Instead, the substrate produces clusters of sharp and intense spots, while misaligned nanoparticles produce arc-shaped signals. The two-dimensional data were integrated over a rectangular sector, chosen to exclude or minimize the contribution of other signals than those coming from the multilayers. Figure S5a shows a representative two-dimensional diffractogram containing signals from the multilayer film, the substrate, and some residual misaligned particles, together with the rectangular sector selected for the integration, shown in yellow.

The instrumental background was measured on a clean silicon wafer and integrated under the same conditions applied for the sample. Then, its 1D profile was modeled into a spline using the MagicPlot software version 2.9.2, and subtracted from the sample diffractogram after being rescaled if needed. A background was considered well-paired with the experimental data if it could adequately describe the diffracted intensity found in regions far from intense multilayer diffraction fringes. Figure S5b shows an example of 1D diffractogram as obtained after the integration of the 2D raw data, together with a properly scaled background ready for the subtraction.

In the event that not all the spurious signals could be excluded during the integration step, a subtraction was attempted. If the extra signal was far from any multilayer fringe, it was simply described as a sum of gaussians, subtracted, and the pattern was locally smoothed by a moving average algorithm. If it was closed or overlapped with the multilayer signal, instead, a different approach was taken. First, the nearby multilayer fringes were fitted with a Gaussian profile to recover their position and broadening. Then, the position and broadening of the overlapping multilayer fringe overlap was inferred from those of the neighboring fringes. Finally, the pattern region affected by the overlap was fitted with the sum of a Gaussian peak, representing the multilayer fringe and having its position and broadening fixed, and a sum of additional peaks describing the additional, unwanted signals. Those were subtracted, and the pattern was then ready for the fit. This data treatment was performed only if the spurious signal was reasonably weak if compared to the neighboring multilayer fringes. If not, the pattern was fitted as it was (as for Figure 3 in the main text), or simply discarded. Figure S5c shows a diffraction pattern after the subtraction of the background and of some spurious signals, shown in red.

The application of the Lorentz Polarization Absorption (LPA) correction is the last treatment performed on the diffractogram before proceeding with the Multilayer Diffraction fit. This step is needed to compensate for the geometrical and instrumental contributions to the diffracted intensity, in order to transform the experimental diffractogram into an measurement of the square modulus of the multilayer structure factor |*F*_*ML*_*(q)*|^2^. This is the mathematical entity computed by our fitting algorithm and directly relatable to the structure of the multilayer. The LPA correction is a function of the beam incidence angle θ and must be applied to each experimental point of the diffractogram and is described by eq 3:^28^
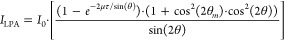
3

In the equation above, *I*_0_ is the intensity measured experimentally (after the background subtraction), μ is the absorption coefficient of the material, τ is the film thickness, and *θ*_*m*_ is the Goebel mirror Bragg angle of the diffractometer (1° in our case). The product *μτ* is responsible for correcting the effects of the X-rays absorption occurring in thick films. However, all our samples belonged to the thin film regime, where the absorption is negligible and *μτ* → 0. Therefore, the product *μτ* was set constant to a small enough value in the fitting algorithm (*μτ* = 0.00001). Smaller values would lead our algorithm to crash. The pattern resulting from the LPA correction is shown in Figure S5d.

### Outline of the Multilayer Diffraction Method

The fitting algorithm presented in this work is an evolution of the one we presented in our previous work on the diffraction analysis of nanocrystal superlattices, to which we refer the reader for additional details.^38^ In short, the algorithm computes the square modulus of the multilayer structure factor |*F*_ML_(*q*)|^2^ based on a structural model of the multilayer, and considers both instrumental and structural parameters. Our algorithm refines these parameters by comparing the simulated |*F*_ML_(*q*)|^2^ with the experimental data processed as illustrated above, *via* the nonlinear least-squares minimization of a cost function. The number and nature of the optimizable parameters was editable, to allow for the maximum flexibility of the code. Those can be divided into three groups, whose impact on the simulation is described below.

The *instrumental parameters* describe the contribution of the diffractometer to the measured diffraction profile. These account for the incident X-ray wavelength, the instrumental broadening of the diffraction signal, the Goebel mirror incidence angle and for possible misalignments of the sample during the measurement. The *multilayer parameters* describe the structure of the multilayer at a superatomic scale. These mostly impact the periodicity and broadening of the diffraction fringes in the diffractogram, and account for the nanoplatelet thickness, the interplatelet distance, the structural disorder of the multilayer, and the electron density of the organic layers in between the nanoplatelets. The *nanoplatelet parameters* describe the atomistic structure of the nanoplatelets. These contribute to the simulation of the nanoplatelet structure factor, and mostly impact the integrated areas of the diffraction fringes in the diffractograms. Their number can be changed to meet the requirements of the specific experiments, and constraints can be imposed during the fit to account for relationships in between them. In general, these parameters describe the vertical coordinate of each atomic layer inside the nanoplatelet, the elements each layer contains and the fractional occupancy of each atom in the layer. A more detailed breakdown of the meaning and role of each parameter is provided in the SI, section S3.

Based on these parameters, the algorithm first computes the structure factors of both the nanoplatelets (*F*_NPL_, by applying the eq 2) and of the interplatelet organic layer (*F*_OL_). The latter is modeled for simplicity as an amorphous layer of carbon atoms, whose density is estimated *a priori* based on the density of long-chain liquid hydrocarbons. For further details see the SI, section S5. The structure factor of the organic layer is computed according to eq 4
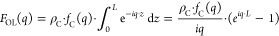
4where ρ_C_ is the linear density of carbon atoms expressed in atoms/Å formula unit, *f*_C_ is the atomic form factor of carbon, and L is the interparticle distance. Once *F*_NPL_ and *F*_OL_ have been calculated, the algorithm proceeds with computing the multilayer structure factor *F*_ML_ first and the multilayer diffraction pattern |*F*_ML_(*q*)|^2^ then, according to the equations derived by Fullerton *et al.* (here not shown for brevity).^28^ As a last step, the algorithm simulates the effects of the instrumental broadening on the just obtained diffraction pattern, and then compares the obtained profile with the experimental one. This comparison is repeated over and over while the program optimizes all the fittable parameters by minimizing the mismatch in between the experimental and the simulated pattern.

To conclude, the values and the standard deviations reported in this article for each parameter are the result of a bootstrap analysis performed on the fit. In short, the algorithm adds to the experimental data set a Gaussian noise estimated over the variance of the experimentally measured diffracted intensity, and then performs the fit. This procedure is repeated 300 times, then the value and standard deviation of each fittable parameter are expressed as the average and standard deviation of the values found at each iteration for said parameter. For further details on the bootstrap procedure and the estimation of the experimental data variance please refer to our previous publication.^38^

### Other Characterization Techniques

The absorption spectra from colloidal suspensions of nanoplatelets were acquired on Cary300 spectrophotometer. The photoluminescence spectra were collected instead on a Cary Eclipse spectrofluorometer. Low magnification transmission electron microscopy (TEM) and selected-area electron diffraction (SAED) images were acquired on a JEOL JEM-1011 microscope equipped with a thermionic gun at an accelerating voltage of 100 kV. The samples were prepared by depositing a diluted suspension of nanoparticles on a 200-mesh carbon-coated copper grids. Energy dispersive X-ray spectroscopy (EDS) measurements were performed at 25 kV on a JEOL JSM-6490LA scanning electron microscope (SEM). X-ray photoelectron spectroscopy XPS measurements were performed with a Kratos Axis Ultra DLD spectrometer. For XPS analysis, high-resolution spectra were acquired at a pass energy of 10 eV using a monochromatic Al Kα source (15 kV, 20 mA). The thickness of iridescent areas of the nanoplatelet films was quantified using a ZETA-20 true color 3D optical profilometer, after the sample surface was softly scratched with a tip of a pair of plastic tweezers. All the crystal structure models presented in this work have been built using VESTA, ver. 3.4.6.^83^

## Supporting Information Available

Experimental conditions for the synthesis of Cs–Pb–X nanoplatelets and detailed synthetic protocol for Cs–Pb–Br nanoplatelet synthesis; description of the background and interferent signals subtraction; outline of the Multilayer Diffraction algorithm; preliminary simulations of the Cs–Pb–X diffraction patterns; parametrization of the Cs–Pb–X nanoplatelet structures and fit results; additional comments on the surface passivation of Cs–Pb–X nanoplatelets; SEM-EDS and XPS compositional analyses of Cs–Pb–X nanoplatelets (PDF)
Experimental XRD diffractograms and corresponding Jupyter Notebooks; Python script containing the Multilayer Diffraction fitting algorithm (ZIP)
