# Supplementary material for: Structure and Surface Passivation of Ultrathin Cesium Lead Halide Nanoplatelets Revealed by Multilayer Diffraction
Source: ACS Nano. 2021 Nov 29;15(12):20341–52. doi: 10.1021/acsnano.1c08636 (PMC8717630; doi:10.1021/acsnano.1c08636)
Supplement: Supplementary file 1 — nn1c08636_si_001.pdf [file nn1c08636_si_001.pdf]

Supporting Information for

Structure and Surface Passivation of Ultrathin Cesium Lead Halide Nanoplatelets Revealed by Multilayer Diffraction

Stefano Toso[1,2,*], Dmitry Baranov[1,*], Cinzia Giannini[3,*], Liberato Manna[1,*]

[1]Nanochemistry Department, Istituto Italiano di Tecnologia, Via Morego 30, 16163, Italy

[2]International Doctoral Program in Science, Università Cattolica del Sacro Cuore, 25121 Brescia, Italy

[3]Istituto di Cristallografia - Consiglio Nazionale delle Ricerche (IC–CNR), Via Amendola 122/O, I-70126 Bari, Italy

*Corresponding authors: stefano.toso@iit.it, dmitry.baranov@iit.it, cinzia.giannini@ic.cnr.it, liberato.manna@iit.it

Contents

S1. Synthesis of Cs-Pb-X nanoplatelets. 2
S2. Diffraction data collection and processing. 6
S3. Multilayer Diffraction pattern simulation. 8
S4. Preliminary Cs-Pb-Br nanoplatelets simulations. 10
S5. Parametrization of the Cs-Pb-Br multilayer and fit results 11
S6. On the surface passivation of Cs-Pb-Br nanoplatelets 14
S7. Preliminary Cs-Pb-I-Cl nanoplatelets simulations. 17
S8. Parametrization of the Cs-Pb-I-Cl multilayer and fit results 18
S9. Simulated patterns for nanoplatelets of different thicknesses 22
S10. References 23

S1. Synthesis of Cs-Pb-X nanoplatelets.

The reproducible synthesis of high-quality colloidal Cs-Pb-Br nanoplatelets is challenging and relies on closely following empirically-developed procedures.[1] Below we describe in detail a typical protocol for synthesis, purification, and thin film preparation for the Cs-Pb-Br nanoplatelets studied in this work. The experimental parameters for other variants (Cs-Pb-Br synthesized in the presence of erucic acid or octylamine, and Cs-Pb-I-Cl nanoplatelets) are summarized in Table S1 and follow similar steps.

Table S1. Reaction conditions adopted for the synthesis of the samples reported in this work.

Sample	Cs-oleate precursor	Pb-carboxylate precursor	Carboxylic acid	Amine	Decane	Benzoyl halides	Reaction Temp.
**Cs-Pb-Br NPLs**	20 µL Cs-oleate	60 µL Pb-oleate	160 µL Oleic acid	120 µL Oleylamine	2 ml	20 µL Bz-Br	100°C
**Cs-Pb-Br NPLs with erucic acid**	25 µL Cs-erucate	75 µL Pb-erucate	172 mg Erucic acid	120 µL Oleylamine	2 ml	20 µL Bz-Br	100°C
**Cs-Pb-Br NPLs with octylamine**	20 µL Cs-oleate	60 µL Pb-oleate	160 µL Oleic acid	60 µL Octylamine	2 ml	20 µL Bz-Br	100°C
**Cs-Pb-I-Cl NPLs**	40 µL Cs-oleate	60 µL Pb-oleate	160 µL Oleic acid	120 µL Oleylamine	2 ml	10+10 µL Bz-Cl + Bz-I	50°C

Preparation of Cs-oleate and Pb-oleate precursor solutions:

In separate 4 ml glass vials, dissolve 192 mg of cesium acetate in 1.0 ml of oleic acid (Cs-oleate precursor), and 379 mg of lead acetate trihydrate in 1.5 ml of oleic acid (Pb-oleate precursor) at ≈120°C under continuous stirring under vacuum. The color of precursors might change from clear colorless to orange-yellow over time. Both precursors solidify upon cooling to room temperature.

Synthesis of Cs-Pb-Br nanoplatelets (1st entry in Table S1):

1. In a 4 ml glass vial equipped with a magnetic stirring bar add 2 ml of decane (measured using a 3 ml disposable syringe), 120 µL oleylamine, 160 µL oleic acid, and heat up under mixing to 100°C (all manipulations were performed in air).
2. Pre-heat Cs-Oleate and Pb-Oleate precursors (not exceeding 100 °C), so they become liquid.
3. Add 20 µL Cs-Oleate and 60 µL Pb-Oleate precursors using a micropipette (as warm/hot liquids, to avoid liquid solidification in the pipette plastic tip). **Note:** The volumes of ligands and metal carboxylates were measured by a single withdrawal of the liquid with a micropipette (using disposable plastic 200 µL micropipette tips) and discharged into the vial with decane following by a repeated rinse of the viscous liquid stuck on the walls of the pipette tip (3-4 times refill/discharge with a pipette) with the hot reaction mixture. This is done to be sure that the transfer of the intended volumes is quantitative; keeping this manner of operation consistent is important for a batch-to-batch reproducibility.
4. After all components were mixed, let the reaction mixture thermalize for a minute.
5. Inject 20 µL of benzoyl bromide using a micropipette under stirring. Dip the tip in the liquid while injecting to be sure that all the benzoyl bromide is transferred into the solution. Do not refill/discharge with a reaction mixture to avoid multiple nucleation events. After the injection of benzoyl bromide, the reaction mixture immediately acquired a clear yellow color (Figure S1a).
6. Let the reaction proceed for 60 seconds.
7. Quench the reaction in a room temperature water bath and proceed with the purification.

Cs-Pb-Br Nanoplatelet Purification and Film Assembly:

8. Precipitate the platelets from the crude reaction mixture with ethyl-acetate. Add slowly until the solution just turns cloudy, followed by a few more droplets (Figure S1b).
9. Centrifuge at 3000 RPM for 2 minutes.
10. Remove the supernatant and repeat the centrifugation with the precipitate pointing outwards. Remove the accumulated liquid (using a cotton tip, a piece of paper tissue or a pipette).
11. Dissolve the remaining solid in 1200 µL of hexanes. The appearance of the solid and the resulting nanoplatelet dispersion are shown in Figure S1(c, d).
12. Centrifuge at 3000 RPM for 2 minutes to remove aggregates.
13. Transfer the liquid (nanoplatelet stock solution) into a clean glass vial (flush with compressed air to remove dust). The quality of the final sample is assessed by measuring its absorption and photoluminescence spectrum. A successful sample shows a sharp excitonic peak without low energy tail or shoulders in the absorption, and a single, slightly asymmetric peak, in the emission (Figure S2).
14. Assemble the film by diluting the nanoplatelet dispersion with hexanes by a factor of 3-to-5 (*e.g.*, 100 µL of the nanoplatelet stock solution plus 400 µL of hexanes for a total dilution by a factor of 5) and depositing 50 µL of it on a 1x1 cm tilted silicon chip (the substrate was tilted about ~15° by putting the small piece of a microscope glass slide with its edge under the center of the silicon chip). The assembly is performed in a closed Petri dish (Figure S3, inset cartoon). A successfully deposited film has an iridescent appearance to a naked eye (Figure S3).

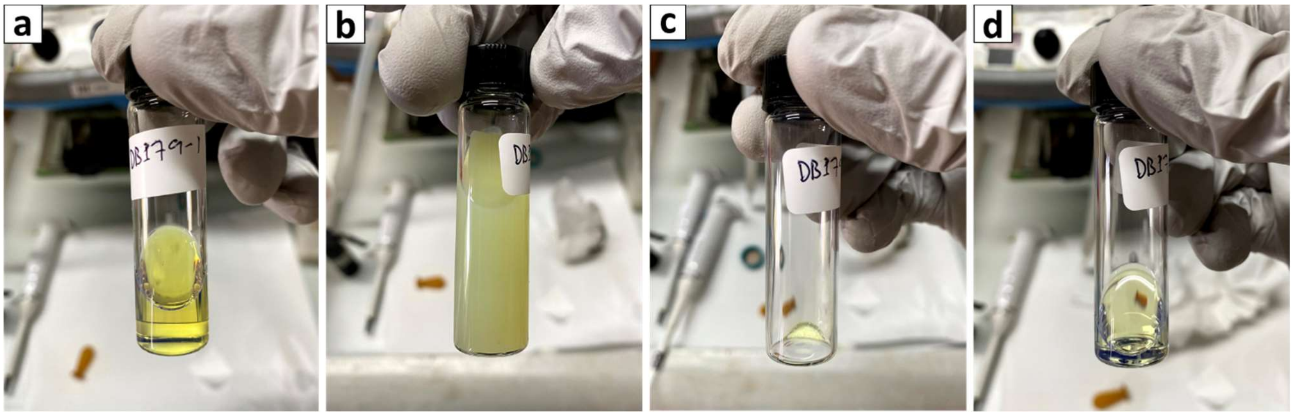

Figure S1. Photographs of the various stages of the Cs-Pb-Br nanoplatelet synthesis and purification: a) clear and yellow reaction mixture after the Bz-Br injection, 1 min growth, and cooling to room temperature in a water bath; (b) cloudy dispersion of flocculated nanoplatelets after the addition of ethyl acetate before the 1st centrifugation; c) precipitated nanoplatelets after the 2nd centrifugation and the removal of residual liquid; d) the isolated solid redispersed in 1.2 ml of hexanes yielding a clear light-yellow solution with a faintly visible blue luminescence caused by ambient light.

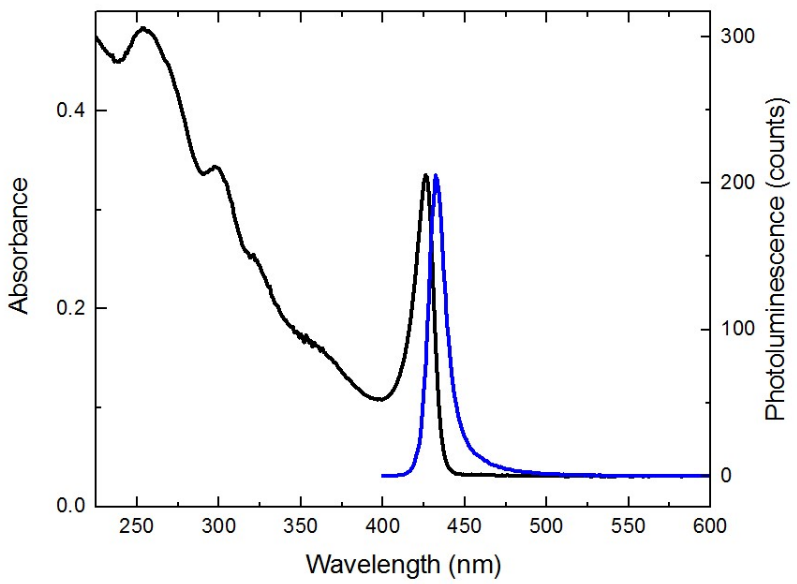

Figure S2. Absorption (solid black curve) and photoluminescence (solid blue curve) of a successful nanoplatelet sample diluted in hexanes. Experimental conditions: 5 µL of the nanoplatelet stock solution diluted in 1 ml of hexanes, optical pathlength 4 mm, λ_{exc} = 350 nm (for emission spectrum).

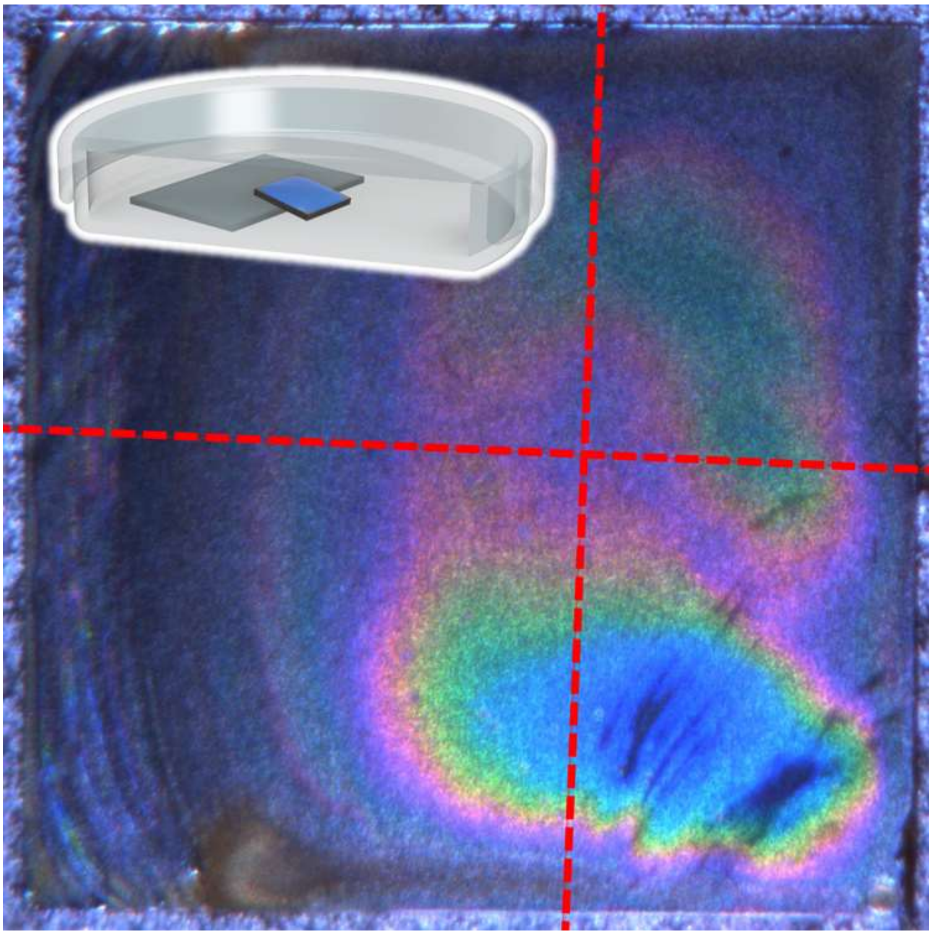

Figure S3. Top view of one sample as seen from the diffractometer automatic alignment system, showing the characteristic iridescence. The dashed red cross shows the spot chosen for the analysis. Inset: graphical representation of the setup used for the self-assembly of thin films.

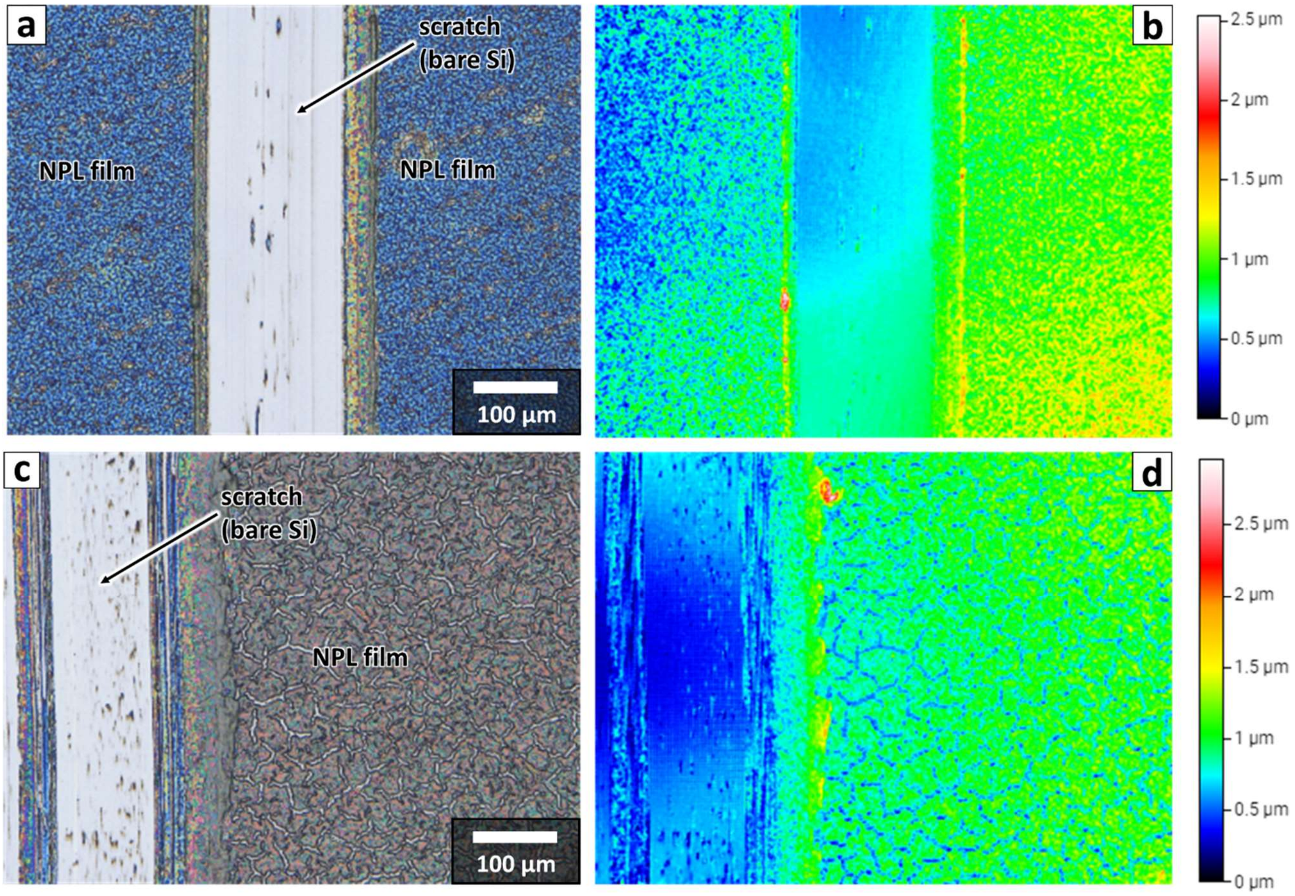

Figure S4. Profilometry RGB (a, c) and height (b, d) images of Cs-Pb-Br nanoplatelet films (a, b) and Ruddlesden-Popper Cs-Pb-Cl-I nanoplatelet films (c, d). The average thickness of the Cs-Pb-Br film in the depicted area is 0.43 ± 0.03 µm (mean ± standard deviation for 10 profile slices), of the Cs-Pb-I-Cl nanoplatelet film: 0.55 ± 0.13 µm (mean ± standard deviation for 10 profile slices). The higher standard deviation for Cs-Pb-I-Cl film is due to the cracks (visible as a blue-colored percolating network in **d**).

S2. Diffraction data collection and processing.

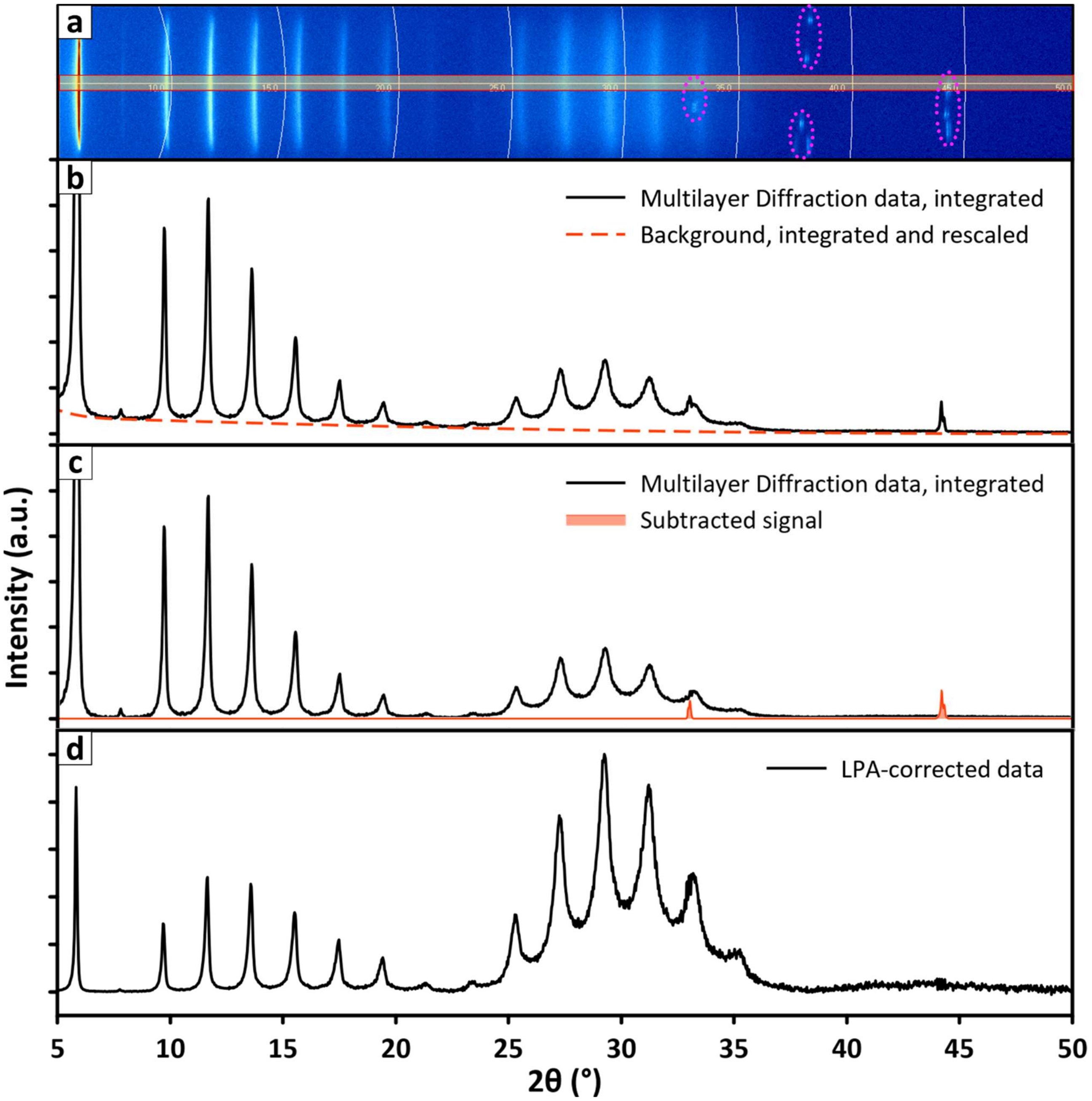

Figure S5. a) 2D-diffraction pattern from a film of nanoplatelets showing the characteristic Multilayer Diffraction periodic reflections. Spurious signals coming from the substrate are encircled. The region of interest for the integration is shaded in yellow. b) 1D pattern obtained after integrating the 2D-data region of interest (shaded in yellow in panel a), shown together with the instrumental background (dashed red line). The instrumental background was measured on a clean silicon wafer, the resulting 2D-diffraction pattern was integrated similarly to the sample, and transformed into a spline for easier data processing. c) Experimental data shown after the subtraction of background and spurious signals (in red). d) Experimental after the background subtraction and the application of the Lorentz Polarization Absorption correction (LPA-correction).

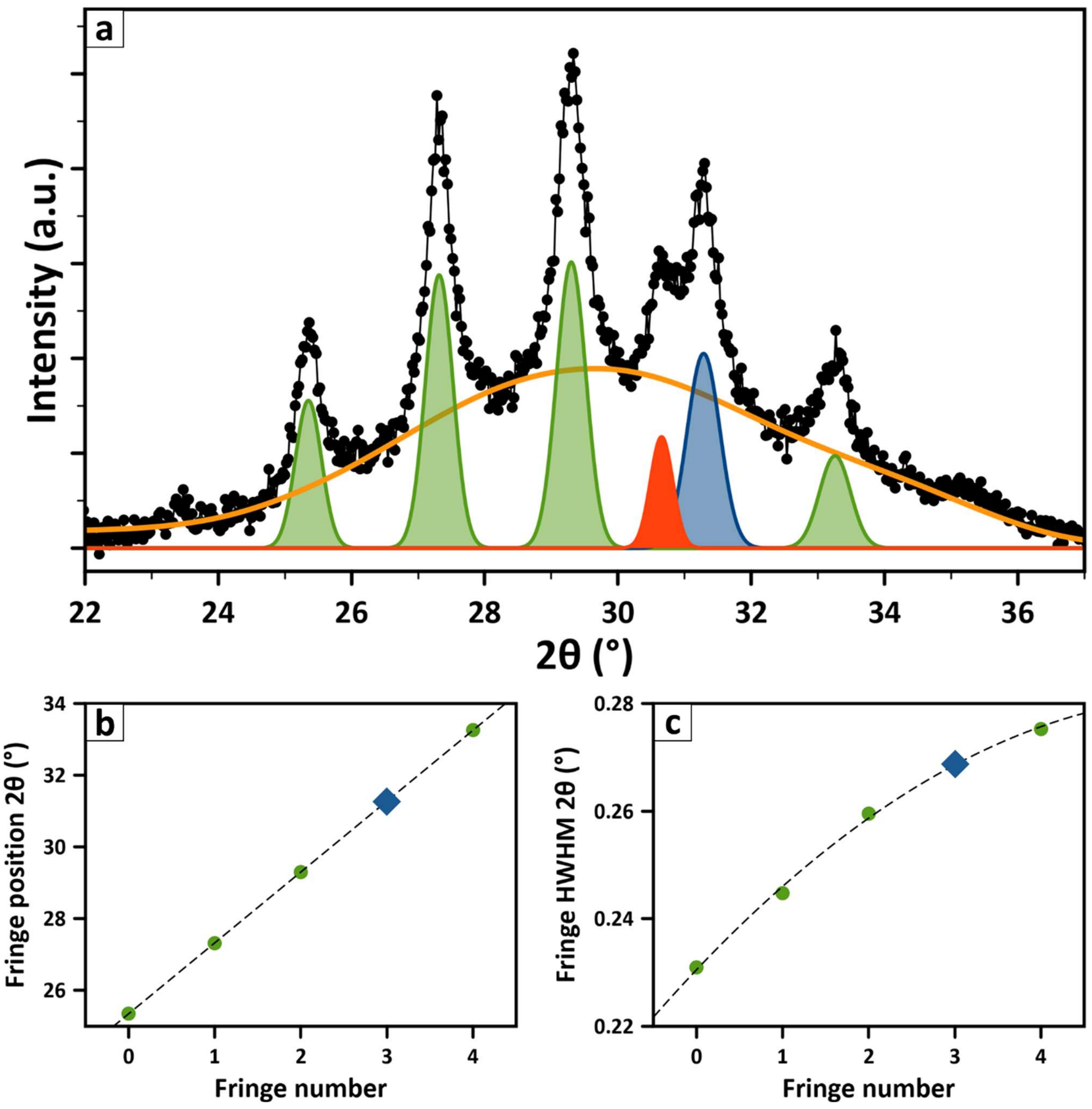

Figure S6. Example of misaligned platelets subtraction. a) To subtract spurious signals overlapping with the multilayer fringes (such as the signal of misaligned platelets, shown in red in this example), the experimental pattern (black markers) is first modelled as the sum of a spline (orange) describing the diffuse scattering and several gaussian functions (green), each one corresponding to a different multilayer fringe that is not affected by the overlap. Then, the position and broadening of the affected fringe (blue) is estimated based on b) the position and c) the broadening of the neighboring fringes. These two values are then fixed for the affected multilayer fringe, and the peak profile of the impurity is recovered by minimizing the differences between the experimental pattern and the model. In this example, the spurious signal from misaligned platelets is found at 2θ = 30.655°, corresponding to a periodicity of 2.917 Å. This corresponds to an in-plane Pb-Pb distance twice as large, equal to 5.833 Å.

S3. Multilayer Diffraction pattern simulation.

Table S2. Summary of the *instrumental* *parameters* in the Multilayer Diffraction algorithm, explaining their origin or physical meaning. If specified, the parameter is refined during the fitting.

**Parameter [symbol]**	**Description and approach to refinement**
**X-ray wavelength [λ_{XRD}]**	Kept fixed at 1.54187 Å (averaged copper Kα1/Kα2).
**Goebel mirror angle**	Bragg angle of the Goebel mirror found in the diffractometer optics, kept fixed at 1° as determined by the actual configuration of the instrument.
**Instrumental broadening [▯$_{Inst}$]**	Broadening of the instrumental response gaussian convolution. It accounts for the broadening of the diffractogram features due to non-ideal response of the instrument. Set free to change during the refinement to better capture the peak shape of the sharp interference fringes found in the multilayer diffractograms. Typical values are in the range 0.01-0.07 $Å^{-1}$.
**Instrumental zero [q_0]**	Correction parameter that shifts the diffractogram in the q-scale by a constant amount. It is used to correct small errors in the diffractometer alignment. Set free to change during the refinement, must be small ($-0.01 < q_0 < 0.01$ $Å^{-1}$)

Table S3. Summary of the *multilayer* *parameters* in the Multilayer Diffraction algorithm, explaining their origin or physical meaning. If specified, the parameter is refined during the fitting.

**Parameter**	**Description and approach to refinement**
**Reference atom-atom length [d]**	A vertical atom-atom distance within the atomistic model of the nanoplatelet that is chosen as a reference to scale all the others. In principle any distance can be chosen, but it is convenient to select one that carries special meaning for the structure. One convenient choice can be the distance between the furthest atoms, that is equal to the nanoplatelet thickness. Another good option is a distance that can be directly compared with the unit cell parameters of the bulk material under investigation, such as the Pb-Pb distance in lead-halide perovskites. Regardless from the choice, the *d* value is refined during the fit.
**Interparticle spacing [L]**	Average thickness of the organic ligands layer in between the nanoplatelets, defined as the vertical distance in between the two closes atoms belonging to different nanoplatelets. Its value mostly depends on the ligands found on the surface of the platelet and is refined during the fit.
**Stacking disorder [▯$_L$]**	Standard deviation of the interparticle spacing, it describes the degree of stacking disorder in the multilayer and is responsible for the broadening of the interference fringes. Its value is ~0.5 Å for the samples studied in this work and is refined during the fit.
**Carbon density [ρ_C]**	Density of carbon atoms in the interparticle spacing region, it is used to approximate the scattering power of the ligands layer. It was kept fixed to a value estimated *a priori* for each sample because it was found to have a negligible effect on our refinements. It might be useful for achieving a better refinement on higher resolution data (*e.g.*, synchrotron).

Table S4. Summary of the *material parameters* in the Multilayer Diffraction algorithm, explaining their origin or physical meaning. If specified, the parameter is refined during the fitting.

Parameter	Description and approach to refinement
**Chemical composition of the atomic planes**	Determines which atomic scattering factors are selected by the algorithm when computing the simulation. It is set before the structural refinement takes place and remains unchanged during the fit.
**Occupancy of atomic sites**	Describes the fractional occupancy of each atomic position. It can be kept fixed or refined, depending on the requirements of the specific experiment. If refined, the value must fall in between 0 (missing atom) and 1 (fully occupied position) to be physically meaningful.
**Fractional z coordinate**	Describes the fractional z-coordinate of each atomic plane inside the nanoplatelet. The product $Z = d \cdot \textit{fractional z coordinate}$ yields the absolute coordinate of an atomic plane within the nanoplatelet. It can be kept fixed or refined depending on the specific experiment.

S4. Preliminary Cs-Pb-Br nanoplatelets simulations.

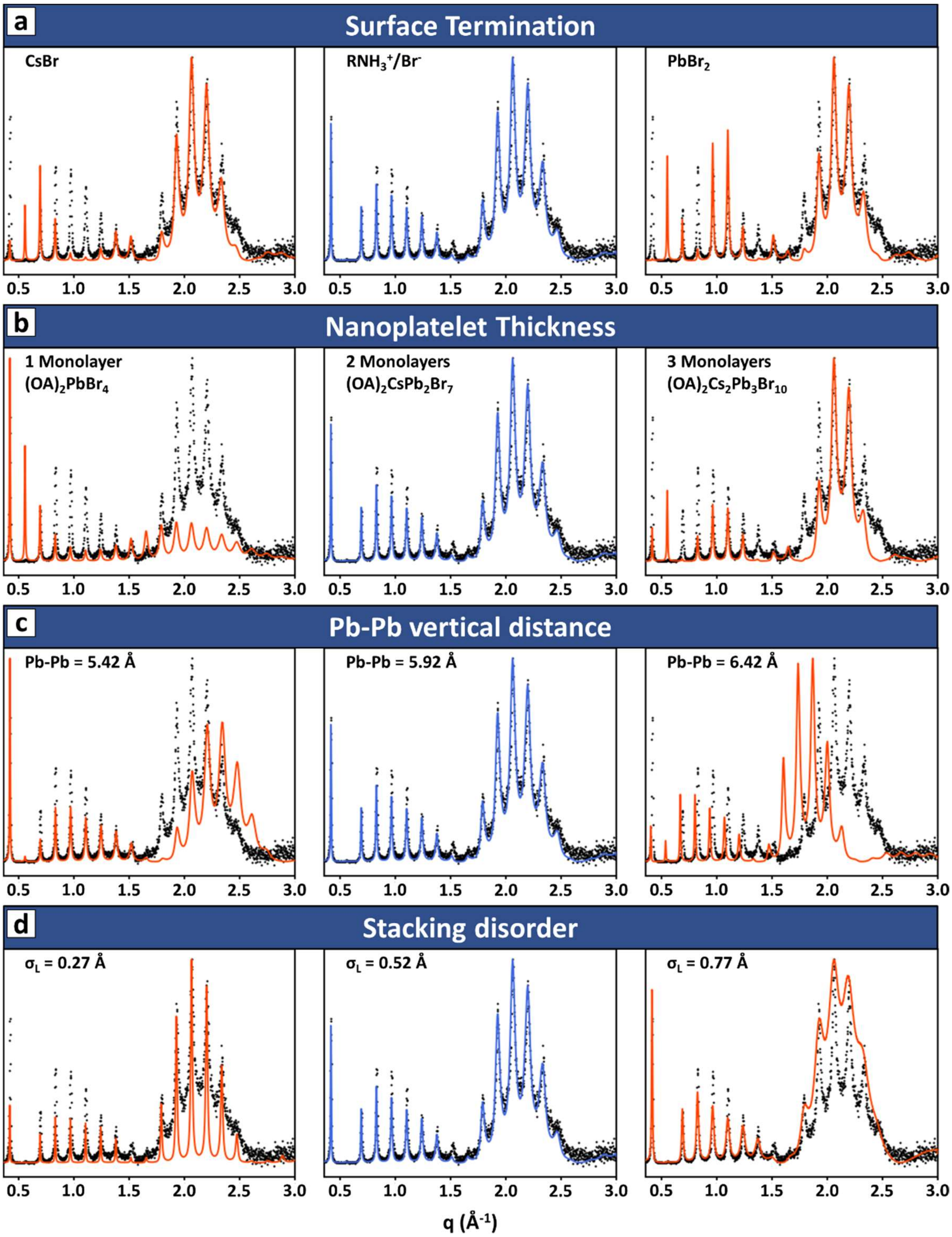

Figure S7. Visual summary of how differences in the structural model of nanoplatelets affect the calculated diffraction pattern. a) Different surface terminations. b) Different nanoplatelet thickness. c) Different Pb-Pb vertical distances, representing the contraction/expansion of the platelet structure along its thinnest direction. d) Stacking disorder. The blue pattern is identical in all the series of simulations, and corresponds to the preliminary best fit (not refining partial occupancies and atomic layer coordinates). The overall multilayer periodicity (*i.e.*, nanoplatelet thickness + interparticle spacing) was kept constant in all the simulations by adjusting the interparticle spacing when needed, in order to ease the comparison.

S5. Parametrization of the Cs-Pb-Br multilayer and fit results

To refine the structure of 2 monolayer Cs-Pb-Br nanoplatelets, their structural model was parametrized as follows. First, the Pb-Pb vertical distance, hereby denoted *d,* was chosen for scaling all the atomic coordinates within the nanoplatelet. This is a convenient choice because it is directly comparable with the pseudocubic unit cell parameter obtained from the orthorhombic unit cell of bulk $CsPbBr_3$. The absolute vertical coordinate of each atomic layer is then expressed as the product $Z = d \cdot \textit{fractional z coordinate}$. The position and content of each atomic layer is then described according to Table S5, which shows the monodimensional representation of the nanoplatelet as fed as an input into the fitting algorithm. The reader may notice that some parameters are fixed (gray tiles), while others are expressed as a function of three independent variables: V_1, V_2, and V_3. These variables are refined during the fitting of the experimental pattern.

V_1 describes the fractional occupancy of the $R\text{-}NH_3^+/Br^-$ surface layers. Here, the hydrogen atoms are omitted due to their negligible scattering power. Note that the occupancy of Br^- and $R\text{-}NH_3^+$ must be set equal to ensure that the multilayer is charge-neutral. Also, it would be impossible to refine the two occupancies independently, because they both contribute to the electron density of the same layer. V_2 describes the fractional occupancy of the CsBr layer in the middle of the nanoplatelet. The occupancy of the $PbBr_2$ layers, here indicated as layers 2+3+4 and 6+7+8, is instead set equal to 1 to serve as a reference for the other two. V_3 instead accounts for the tilting of the octahedra in the structure. If $V_3 = 0$ the atomic layers 2+3+4 and 6+7+8 merge into two flat $PbBr_2$ layers, and the structure turns from orthorhombic ($|V_3| > 0$) into tetragonal.

Table S5. Parametrization of the structure of 2 monolayer Cs-Pb-Br nanoplatelets.

Atomic layer N°	Fractional Z coordinate	Element 1	Occupancy 1	Element 2	Occupancy 2
**1**	2	N	V_1	Br^-	V_1
**2**	$1.5 + V_3$	Br^-	1		
**3**	1.5	Pb^{2+}	1	Br^-	1
**4**	$1.5 + V_3$	Br^-	1		
**5**	1.0	Cs^+	V_2	Br^-	V_2
**6**	$0.5 + V_3$	Br^-	1		
**7**	0.5	Pb^{2+}	1	Br^-	1
**8**	$0.5 - V_3$	Br^-	1		
**9**	0	N	V_1	Br^-	V_1

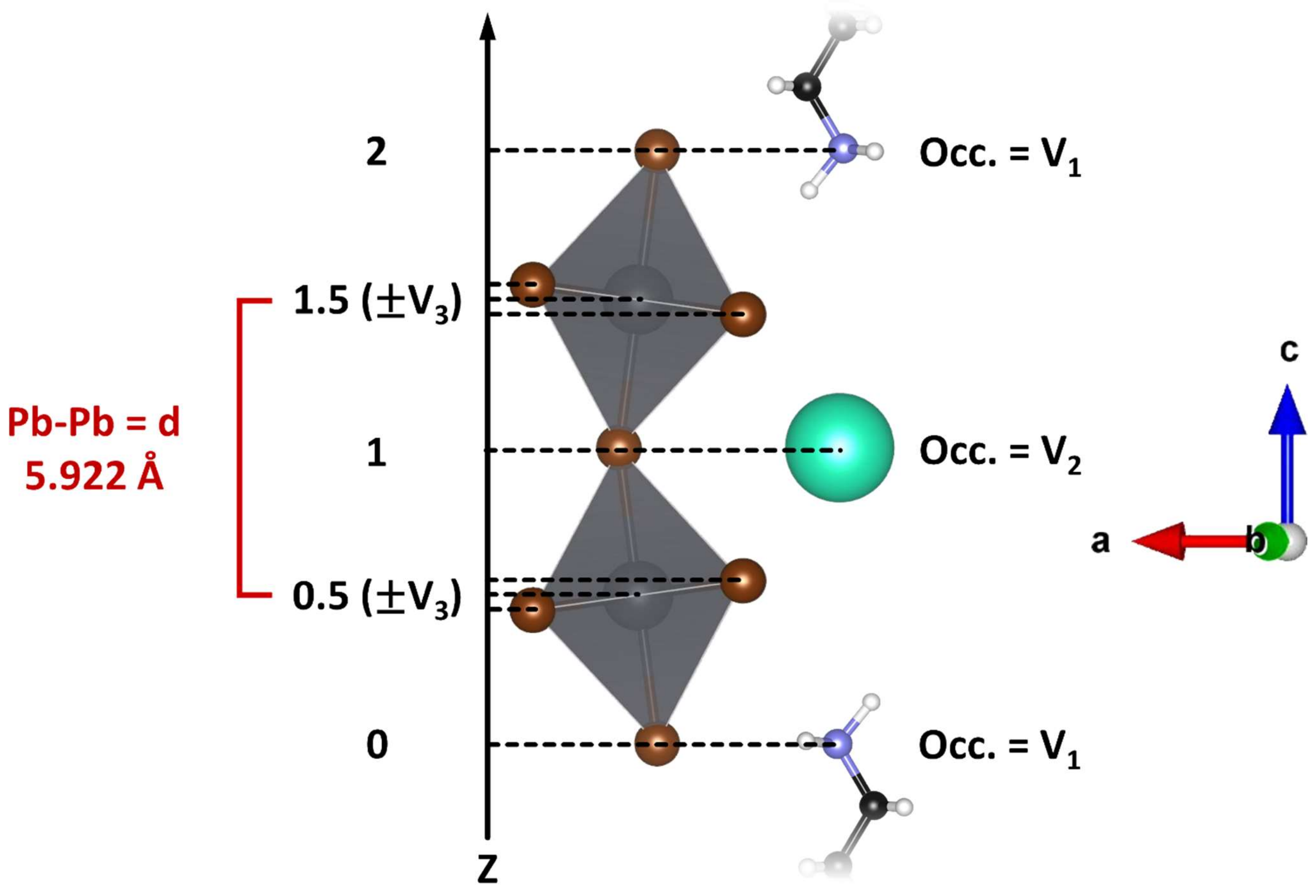

Figure S8. Visual representation of Table S5, showing how the one-dimensional representation of the nanoplatelet relates with the three-dimensional crystal structure of the nanoplatelet.

The interparticle spacing, containing the aliphatic chains of the oleylammonium ligands, was instead described as a homogeneous layer of amorphous carbon having a one-dimensional carbon atoms density of ρ_C = 1.15 *C atoms / Å · formula unit*. This value was estimated as follows. First, we considered the density of 1-octadecene, which is the hydrocarbon most similar to the aliphatic chains of oleylamine (ρ_{ODE} = 0.789 g/cm^3). Second, we converted this value to the volume density of carbon atoms, 0.0339 *Carbon atoms / Å^3,* by using the molecular weight, Avogadro's number, and number of carbon atoms per molecule of 1-octadecene. We then assumed that the space in between particles was filled with identical density. Next, we had to translate this value into a linear density expressed as *C atoms / Å · formula unit*. This is needed to ensure that only two oleylammonium ligands (top and bottom surface) were present for each passivation site on the surface of the platelets, and that the electron-neutral $(NH_3\text{-}R)_2CsPb_2Br_7$ composition of the platelets was correctly represented by our model. To do so, we measured the horizontal (in-plane xy) Pb-Pb distance by exploiting the peaks coming from misaligned platelets in the sample (Pb-Pb_{hor} = 5.833 *Å*) and calculated the area associated with each docking site of $R\text{-}NH_3^+$ on the nanoplatelet surface (Pb-Pb_{hor}^2 = 34.02 *Å^2*). This area corresponds exactly to one each $[CsPb_2Br_7]^{2-}$ formula unit of the inorganic nanoplatelet. Multiplying this area by the volume density of carbon atoms we convert the interparticle volume from *Å^3* to *Å · formula unit*, and obtain the one-dimensional carbon atoms density as 1.15 *C atoms / Å · formula unit*.

Once both the structure of the nanoplatelets and that of the interparticle organic layers were described we fed them into the fitting algorithm, which optimized 9 fittable parameters: d, L, $\square_L$, q_0, $\square_{Inst}$, ρ_C, V_1, V_2, V_3. For the description of fittable parameters please refer to Section S3. Figure S9 summarizes the results of the fit after the bootstrap analysis (N=300, implementation of this analysis has been described previously).[2] The diagonal elements show the distribution of fitted parameters for each bootstrap iteration, from which the average value (black dashed lines) and the standard deviation (red dotted lines) are extracted. The off-diagonal elements represent instead the cross-correlations between two different parameters of the model. The presence of twin spots in the cross-correlation maps indicates two local minima close to each other in the space of parameters. However, both were within the range of standard deviation for all the variables, and converge into one single set of physically meaningful best values.

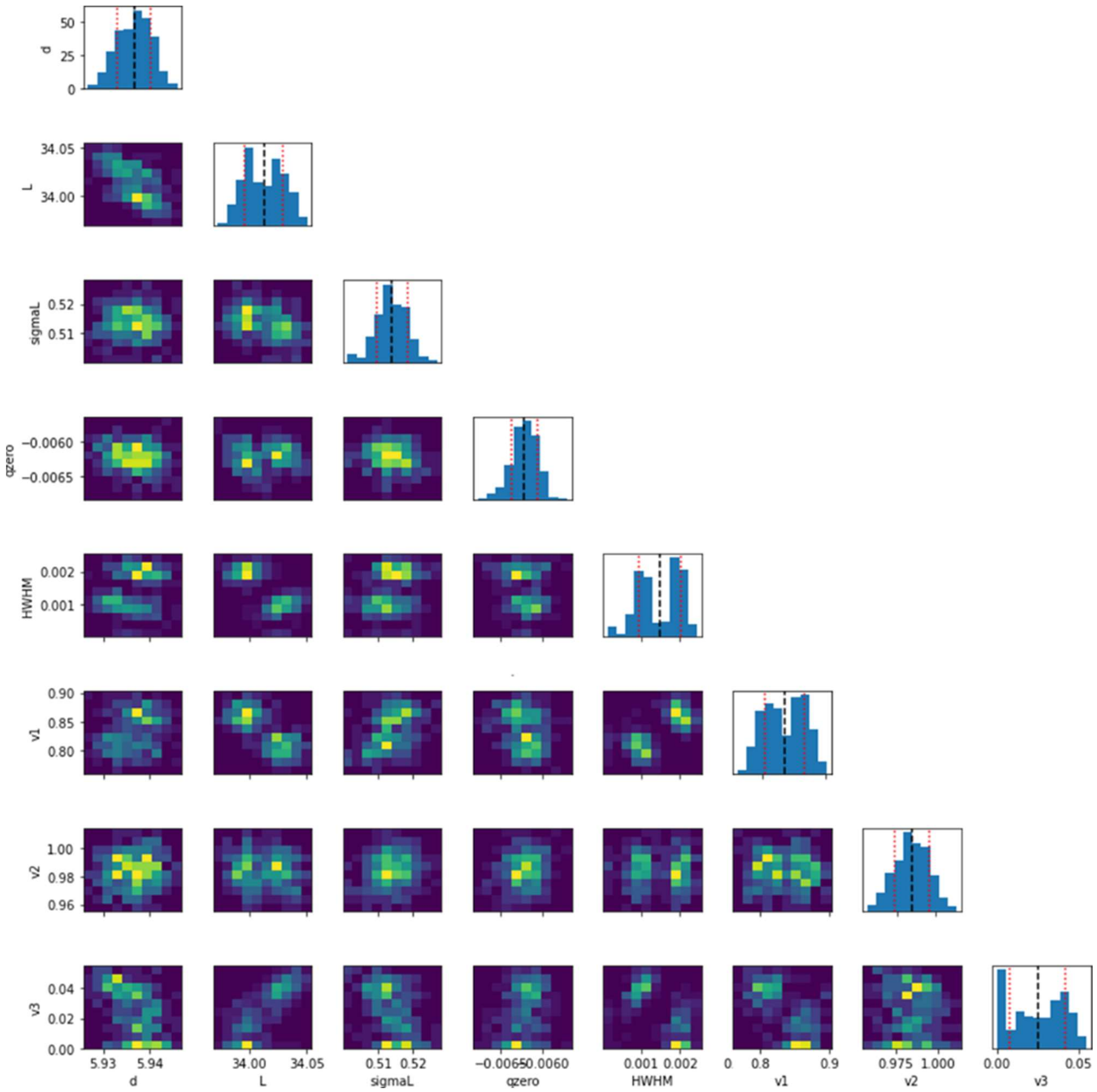

Figure S9. Results of the bootstrap analysis on the two monolayer Cs-Pb-Br nanoplatelets fit, showing the distribution of the best-fit parameters for each iteration of the bootstrap procedure (diagonal elements) and the cross-correlations between pairs of parameters (off-diagonal elements).

S6. On the surface passivation of Cs-Pb-Br nanoplatelets

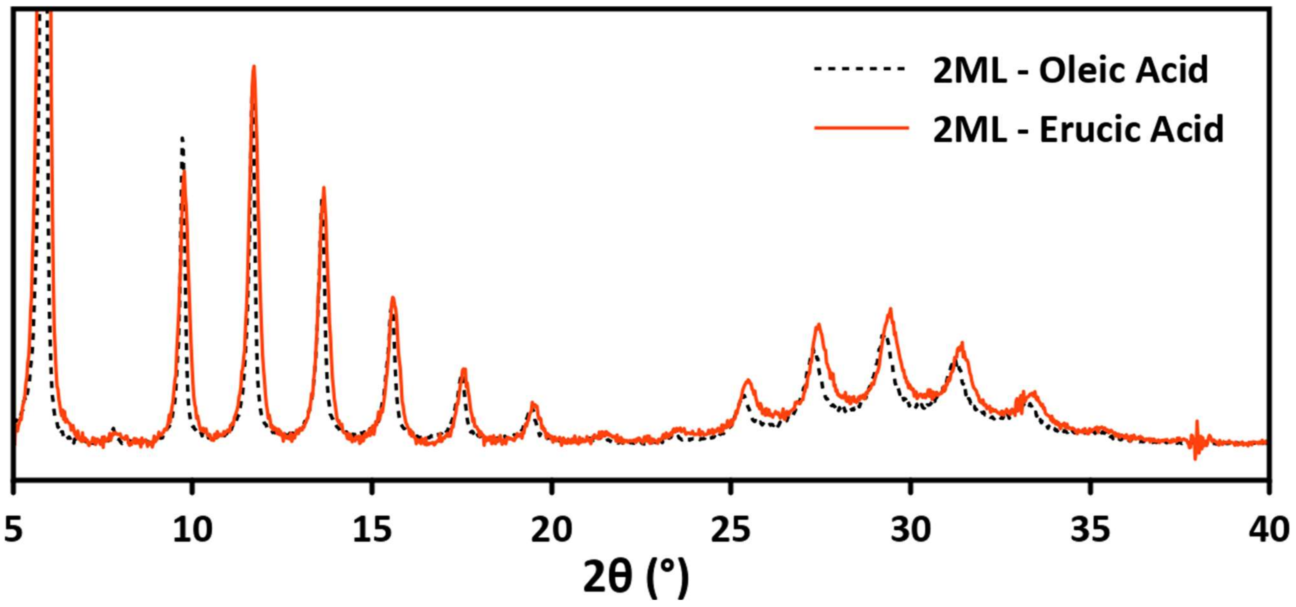

Figure S10. Diffraction patterns of 2 monolayer Cs-Pb-Br nanoplatelets samples prepared with oleic acid (black dashed line) and with erucic acid (red solid line) compared. The patterns are shown after the background and spurious signal subtraction, but before the application of the LPA correction. It is clearly visible that the spacing between diffraction fringes has been little affected by the longer acid (see fit results for erucic acid sample in Figure S11, and contrast with the effect of octylamine in Figures S12 and S13).

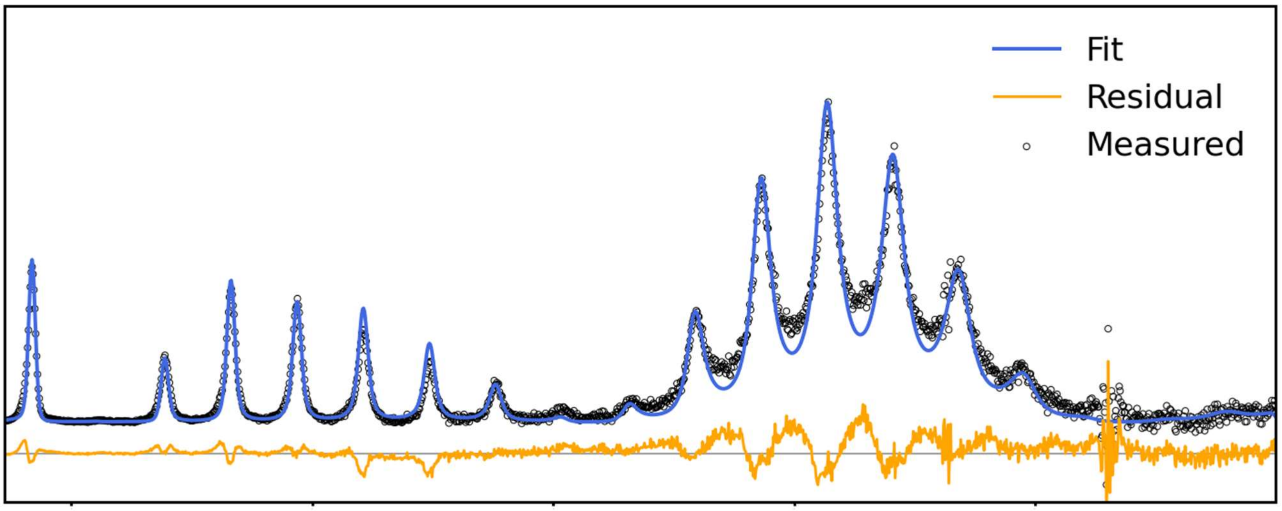

Figure S11. Best fit for the Cs-Pb-Br nanoplatelets sample prepared with erucic acid. The best fit parameters are comparable within error to what measured on samples prepared with oleic acid: Pb-Pb distance = 5.931 ± 0.006 Å; L = 33.84 ± 0.02 Å; σ_L= 0.491 ± 0.008 Å; surface occupancy = 74 ± 3 %; CsBr layer occupancy = 94 ± 2 %; $|z_{Pb}-z_{Br}|$ = 0.29± 0.06 Å.

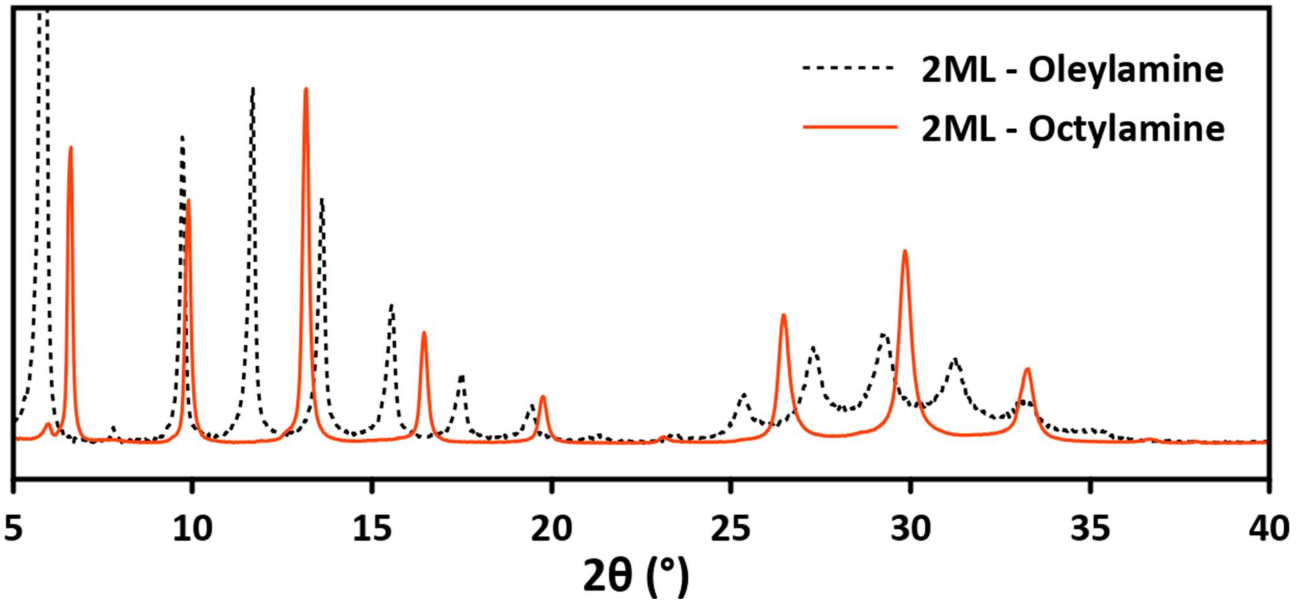

Figure S12. Diffraction patterns of 2 monolayer Cs-Pb-Br nanoplatelets samples prepared with oleylamine (black dashed line) and with octylamine (red solid line) compared. The patterns are shown after the background and spurious signal subtraction, but before the application of the LPA correction. It is clearly visible by eyes that the diffraction fringes are more spaced (= shorter interparticle distance) and sharper (lower stacking disorder) in the case of octylamine.

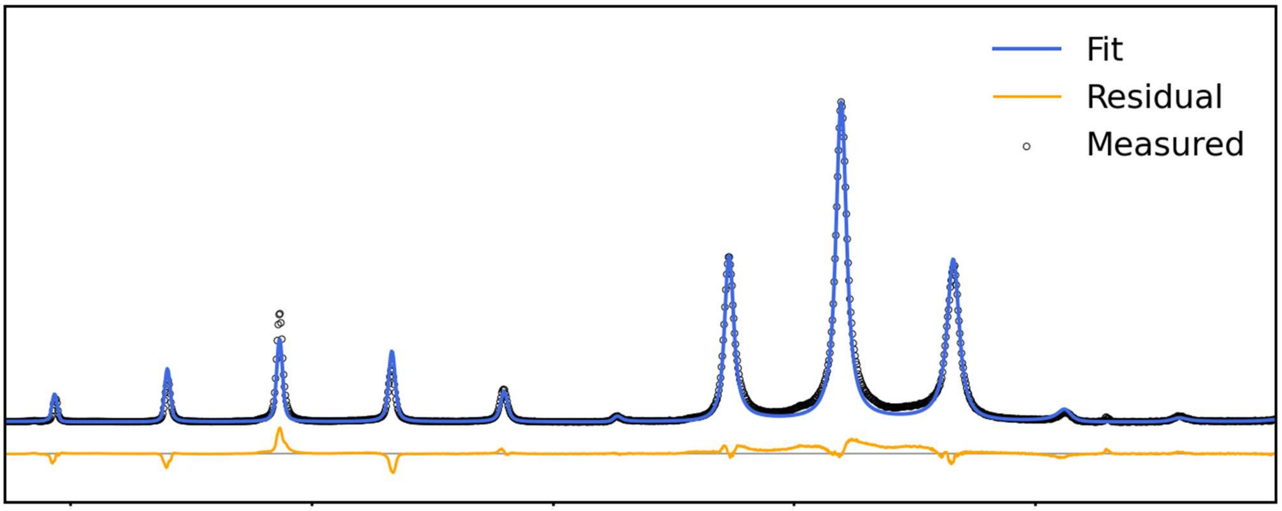

Figure S13. Best fit for the Cs-Pb-Br nanoplatelets sample prepared with octylamine. The fit correctly captures the shorter interparticle distance and the lowered stacking disorder expected as a consequence of using shorter ligands: Pb-Pb distance = 5.90 ± 0.01 Å; L = 15.16 ± 0.03 Å; σ_L= 0.240 ± 0.006 Å; surface occupancy = 87 ± 4 %; CsBr layer occupancy = 0.96 ± 0.02 %; $|z_{Pb}$-$z_{Br}|$ = 0.32± 0.05 Å.

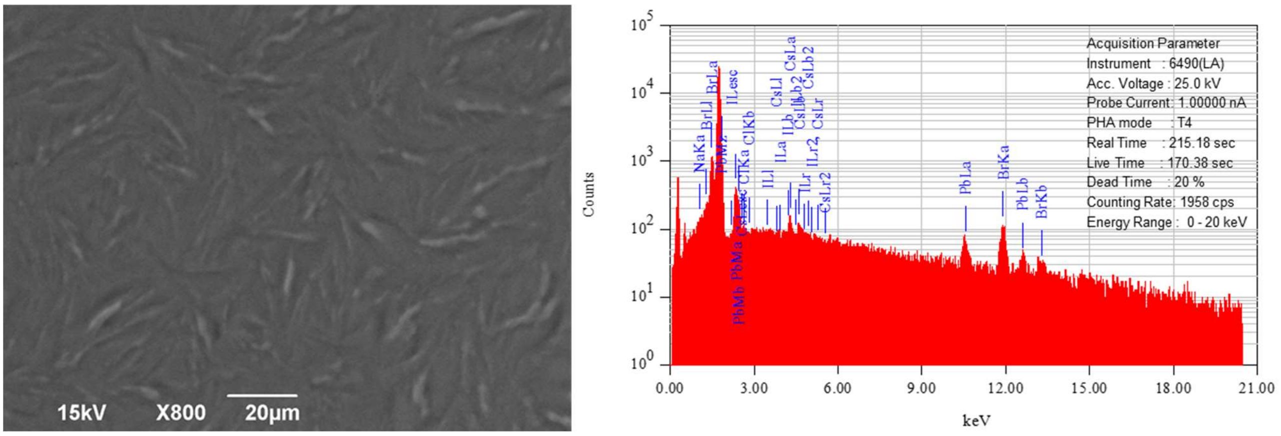

Figure S14. A representative SEM image (left panel, acquired at 15 kV) and EDS spectrum (right panel, acquired at 25 kV) of a Cs-Pb-Br nanoplatelets sample that has been analyzed with Multilayer Diffraction.

Table S6. Results of the SEM-EDS compositional analysis on Cs-Pb-Br nanoplatelets. Reported values are atomic %. The ratio column is normalized to Pb = 2 for a comparison with predicted stoichiometries (vacancy-free Cs : Pb : Br = 1 : 2 : 7 , with vacancies from Multilayer Diffraction fit Cs : Pb : Br = 0.95 : 2 : 6.41).

	Region 1	Region 2	Region 3	Region 4	Average	St. Dev.	Ratio
**Cs**	8.96	10.68	9.87	10.29	9.95	0.74	0.93
**Pb**	22.53	21.28	21.07	20.58	21.37	0.83	2
**Br**	68.51	68.04	69.06	69.14	68.69	0.51	6.43

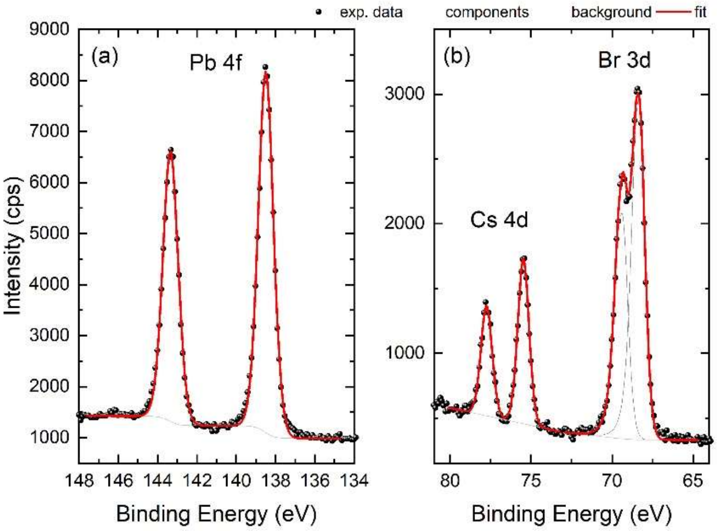

Figure S15. XPS spectra collected on the Cs-Pb-Br nanoplatelets sample. (a) Pb 4f peaks; (b) Cs 4d and Br 3d peaks. Experimental data (black circles) and the best fits (continuous red line) are shown.

Table S7. Results of the XPS compositional analysis on Cs-Pb-Br nanoplatelets. Reported values are averaged atomic % collected from a 300 x 700 μm area of the sample.

	Region 1	Ratio
**Cs**	10.97	1
**Pb**	21.86	2
**Br**	67.17	6.1

S7. Preliminary Cs-Pb-I-Cl nanoplatelets simulations.

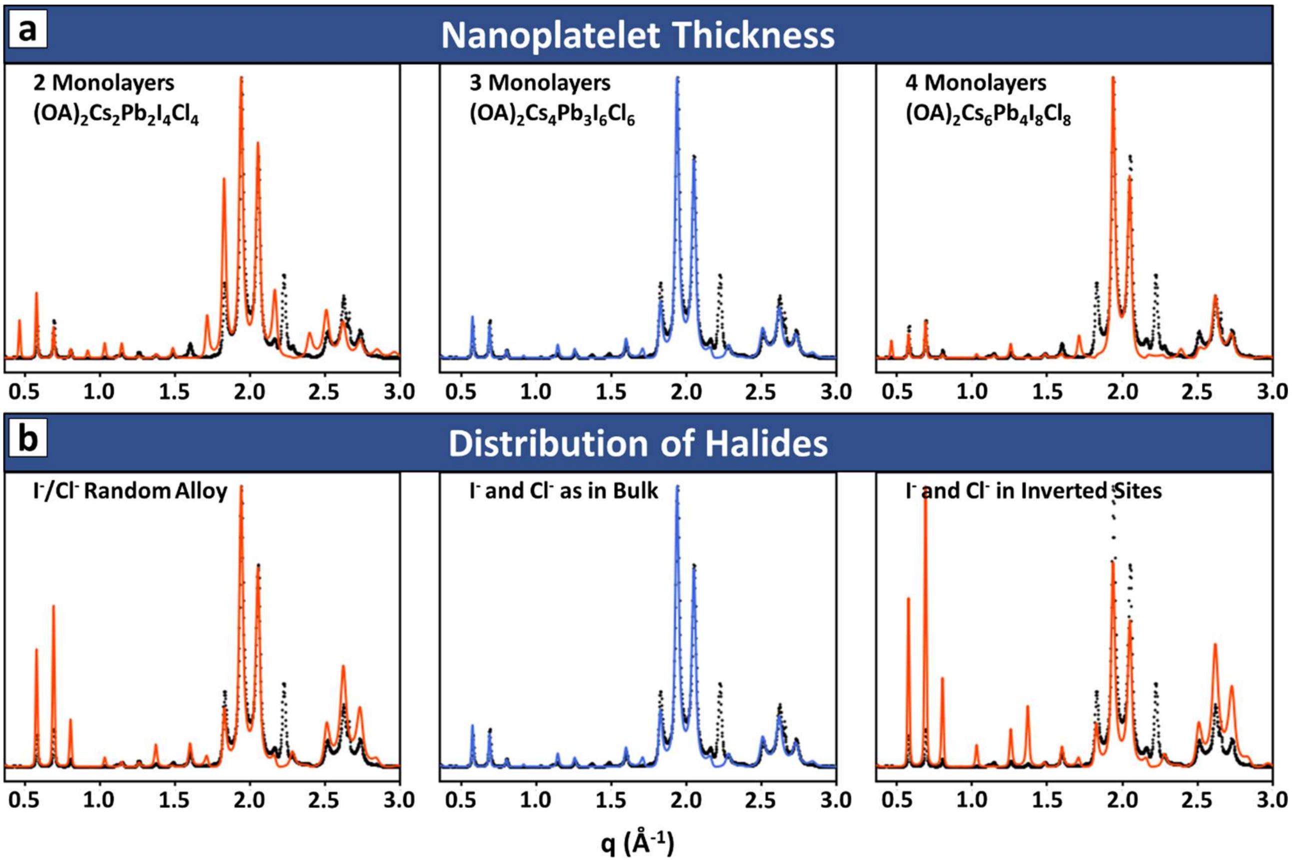

Figure S16. Preliminary simulations based on the bulk structure of bulk $Cs_2PbI_2Cl_2$, performed to identify a) the thickness of nanoplatelets and b) the distribution of Cl^- and I^- inside the structure. The only model that qualitatively captures the intensity of multilayer fringes is an oleylammonium-iodide terminated, 3 monolayers thick Ruddlesden-Popper nanoplatelet (corresponding to a $(OA)_2Cs_4Pb_3I_6Cl_6$ stoichiometry), in which the Cl^- and I^- ions occupy separate crystallographic sites as in the published bulk structure. This model was therefore selected as the starting point for the refinement of the nanoplatelets structure.

S8. Parametrization of the Cs-Pb-I-Cl multilayer and fit results

To refine the structure of 3 monolayer Cs-Pb-I-Cl nanoplatelets, their structural model was parametrized as follows. First, the vertical distance between the two most distant Pb-Pb atoms, hereby denoted *d,* was chosen for scaling all the atomic coordinates within the nanoplatelet. This is a convenient choice because it is directly comparable with the *c* unit cell parameter of bulk $Cs_2PbI_2Cl_2$. The absolute vertical coordinate of each atomic layer is then expressed as the product *Z = d · fractional z coordinate.* The position and content of each atomic layer was described according to Table S8, which shows the monodimensional representation of the nanoplatelet as fed as an input for our algorithm. Again, some parameters were fixed (gray tiles), while others were expressed as a function of three independent variables: V_1, V_2, and V_3. These variables are refined during the fitting of the experimental pattern.

V_1 describes the fractional occupancy of the R-NH_3^+/I^- surface layers. V_2 describes the average z coordinate of the Cs^+/I^- atomic planes, while V_3 represents the misplacement of both the Cs^+ and the I^- atomic planes from their ideal common position along *z*, accounting for the wrinkling of the Cs^+/I^- layer.

Table S8. Parametrization of the structure of 3 monolayer Cs-Pb-Cl-I nanoplatelets.

Atomic layer N°	Fractional Z coordinate	Element 1	Occupancy 1	Element 2	Occupancy 2
1	$1 + V_2 + V_3$	I^-	V_1	N	V_1
2	1	Pb^{2+}	1	Cl^-	2
3	$1 - V_2 + V_3$	Cs^+	1		
4	$1 - V_2 - V_3$	I^-	1		
5	$0.5 + V_2 + V_3$	I^-	1		
6	$0.5 + V_2 - V_3$	Cs^+	1		
7	0.5	Pb^{2+}	1	Cl^-	2
8	$0.5 - V_2 + V_3$	Cs^+	1		
9	$0.5 - V_2 - V_3$	I^-	1		
10	$V_2 + V_3$	I^-	1		
11	$V_2 - V_3$	Cs^+	1		
12	0	Pb^{2+}	1	Cl^-	2
13	$- V_2 - V_3$	I^-	V_1	N	V_1

The interparticle spacing, containing the aliphatic chains of the oleylammonium ligands, was described as a homogeneous layer of amorphous carbon having a linear carbon density of ρ_C = 1.09 *C atoms / Å · formula unit*. This value was obtained by applying the same method described in the section S5 above.

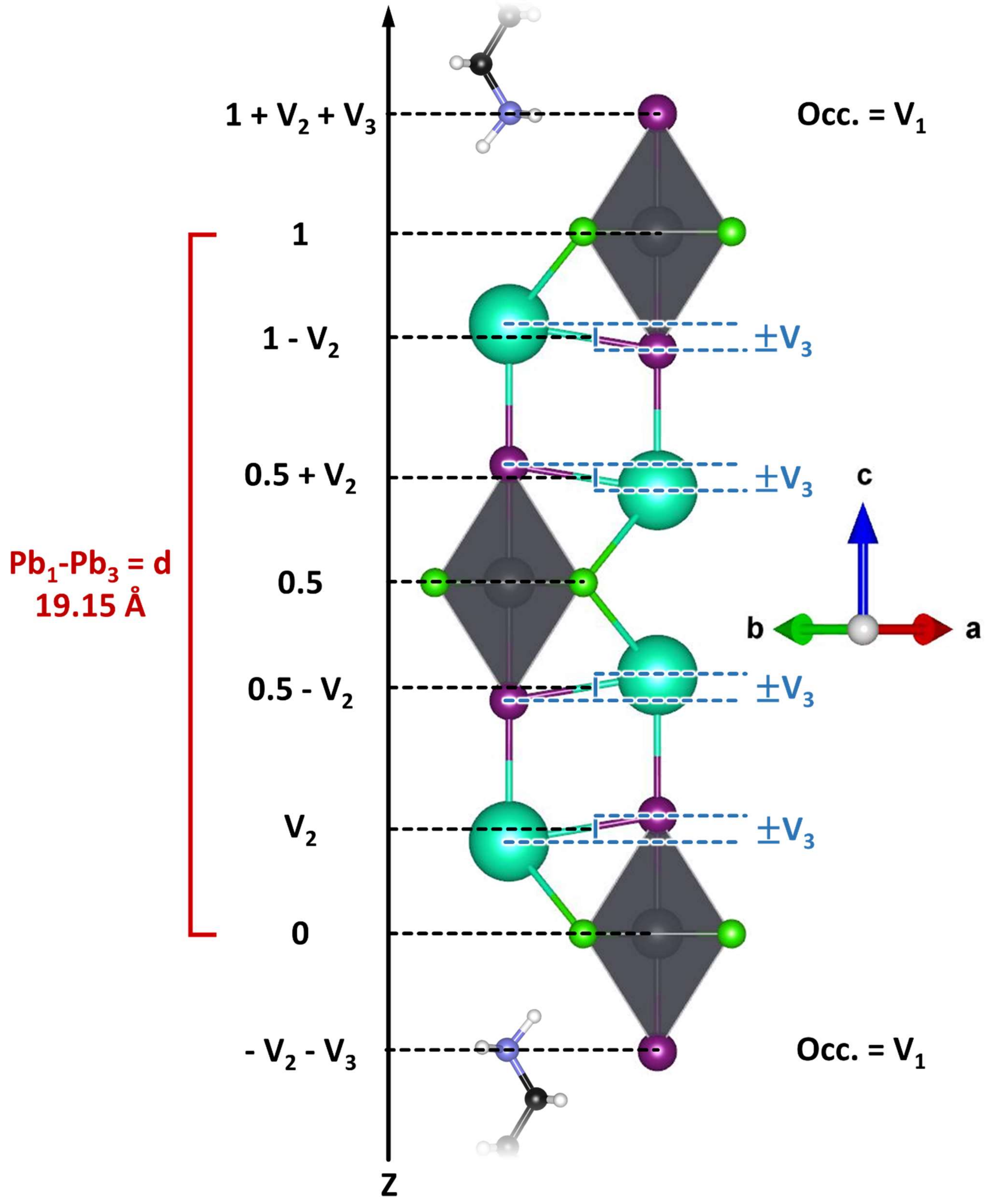

Figure S17. Visual representation of Table S8, showing how the one-dimensional representation of the nanoplatelet relates with the three-dimensional structure of the nanoplatelet.

Once both the structure of the nanoplatelets and that of the interparticle organic layers were described we fed them into the fitting algorithm, which optimized 8 fittable parameters: *d*, *L*, □$_L$, q_0, □$_{Inst}$, ρ_C, V_1, V_2, V_3. For the description of fittable parameters please refer to Section S3. Figure S18 summarizes the results of the fit after the bootstrap analysis (N=300, implementation of this analysis has been described previously).[2] The diagonal elements show the distribution of fitted parameters for each bootstrap iteration, from which the average value (black dashed lines) and the standard deviation (red dotted lines) are extracted. The off-diagonal elements represent instead the cross-correlations between two different parameters of the model. Most of the parameters show little-to-no sign of cross-correlation, as demonstrated by the round-shaped spots in the cross-correlation maps. The only exception is v3, which partially correlates with *d* and *L* (and therefore to the overall multilayer periodicity). Nevertheless, the resulting standard deviation for v_3 is small enough to demonstrate that the Cs^+ and I^- ions in nanoplatelets are better aligned than in bulk $Cs_2PbI_2Cl_2$.

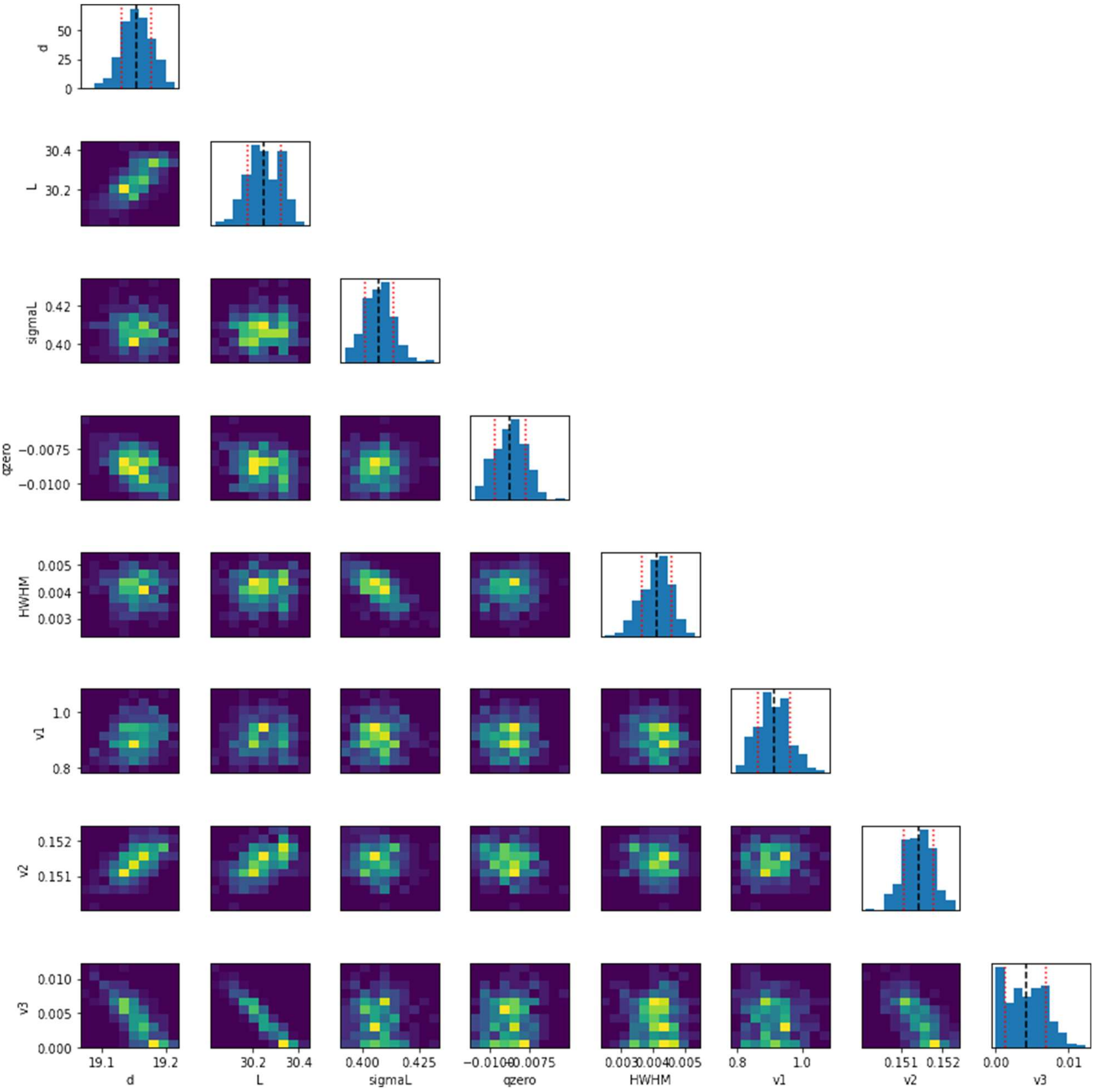

Figure S18. Results of the bootstrap analysis on the two monolayer Cs-Pb-I-Cl nanoplatelets fit, showing the distribution of the best-fit parameters for each iteration of the bootstrap procedure (diagonal elements) and the cross-correlations between pairs of parameters (off-diagonal elements).

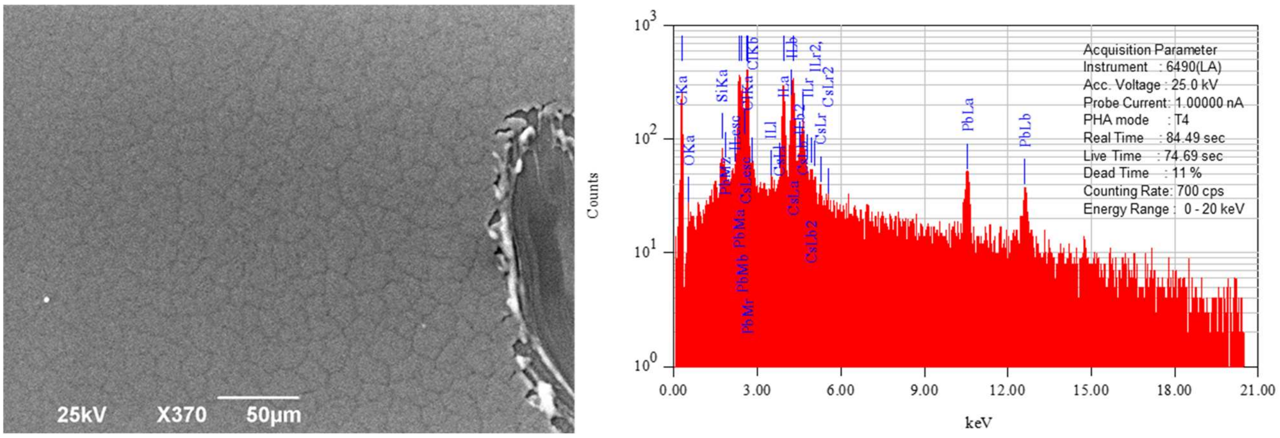

Figure S19. A representative SEM image (left panel, the uniformly flat film was intentionally scratched on the right side of the image to provide contrast with the substrate) and EDS spectrum (right panel) of a Cs-Pb-I-Cl nanoplatelets sample that has been analyzed with Multilayer Diffraction.

Table S9. Results of the SEM-EDS compositional analysis on Cs-Pb-I-Cl nanoplatelets. Ratio is normalized to I = 5.8 for a comparison with a stoichiometry predicted from XRD fit (Cs : Pb : I : Cl = 4 : 3 : 5.8 : 6).

	Region 1	Region 2	Region 3	Region 4	Average	St. Dev.	Ratio
**Cs**	19.92	20.19	18.67	20.77	19.89	0.89	4.8
**Pb**	16.90	17.80	21.84	15.08	17.91	2.86	4.3
**I**	25.43	25.25	21.86	23.58	24.03	1.67	5.8
**Cl**	37.75	36.75	37.63	40.57	38.18	1.66	9.2

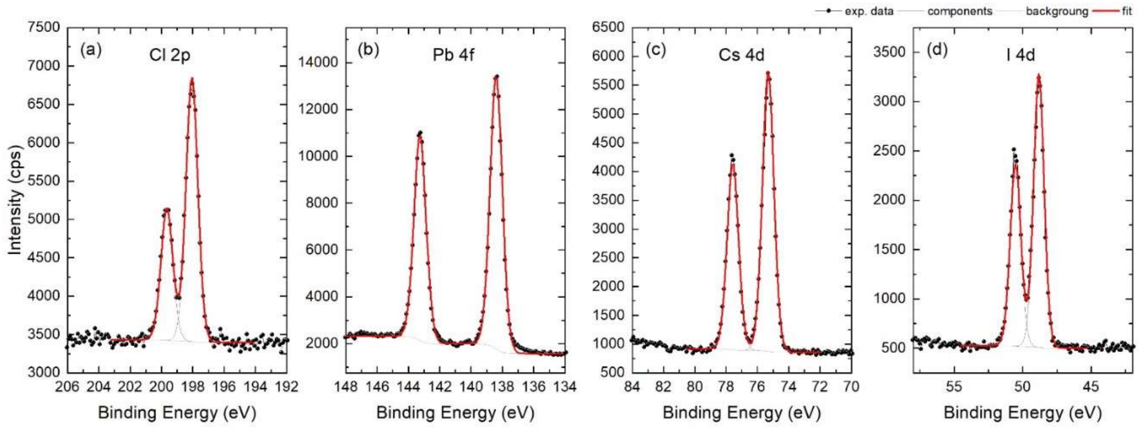

Figure S20. XPS spectra collected on the Cs-Pb-I-Cl nanoplatelets sample. (a) Cl 2p peaks; (b) Pb 4f peaks; (c) Cs 4d peaks; (d) I 4d peaks. Experimental data (black circles) and the best fits (continuous red line) are shown.

Table S10. Results of the XPS compositional analysis on Cs-Pb-I-Cl nanoplatelets. Values reported in the first column are average atomic %. The ratio column is normalized to I = 5.8 for a comparison with a stoichiometry predicted from XRD fit (Cs : Pb : I : Cl = 4 : 3 : 5.8 : 6).

	Region 1	Ratio
**Cs**	22.83	7
**Pb**	18.39	5.6
**I**	18.92	5.8
**Cl**	39.86	12.2

S9. Simulated patterns for nanoplatelets of different thicknesses

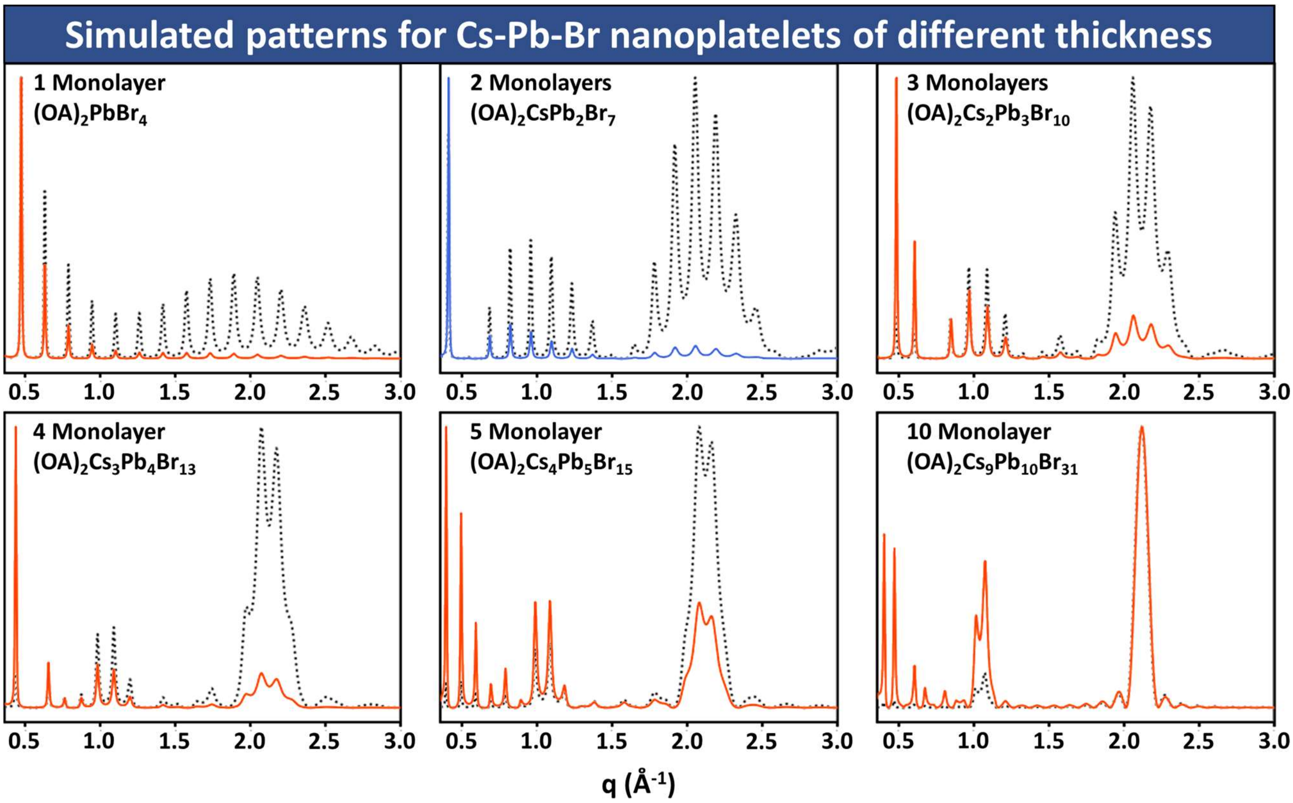

Figure S21. Simulated diffraction patterns for Cs-Pb-Br nanoplatelets of different thicknesses. The solid-colored lines show the expected as-measured diffraction patterns before the application of LPA correction; the LPA-corrected patterns are instead shown as dashed black lines. The simulations were performed by keeping the same interparticle spacing, surface passivation and degree of octahedral tilting as measured on the 2ML Cs-Pb-Br nanoplatelets, to provide a realistic simulation. The stacking disorder parameter was instead changed linearly to account for the higher disorder expected for thicker nanoplatelets. As reference values we chose 2ML = 0.5 Å (as measured in this work on Cs-Pb-Br nanoplatelets) and 13ML = 1.4 Å (as measured in Ref. 2 on $CsPbBr_3$ nanocrystal superlattices).

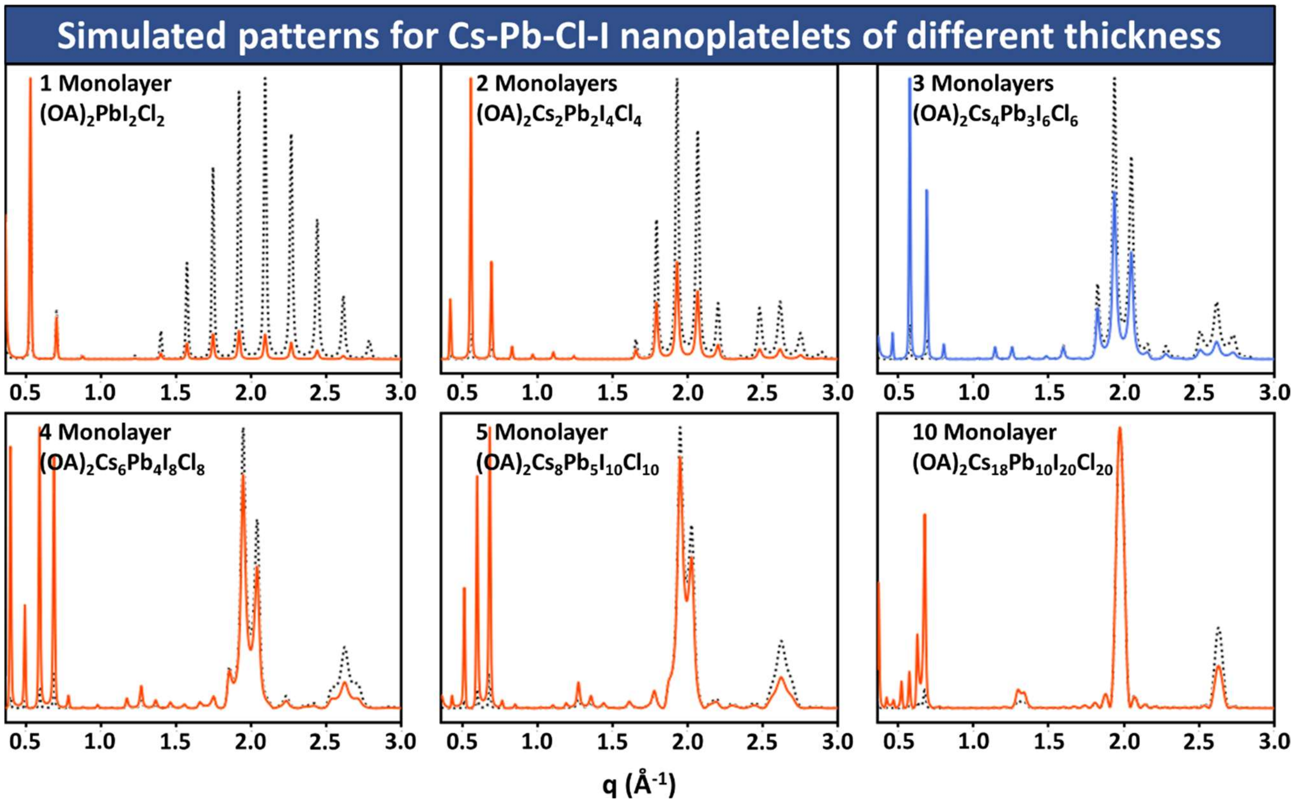

Figure S22. Simulated diffraction patterns for Cs-Pb-I-Cl nanoplatelets of different thicknesses. The solid-colored lines show the expected as-measured diffraction patterns before the application of LPA correction; the LPA-corrected patterns are instead shown as dashed black lines. The simulations were performed by keeping the same interparticle spacing, surface passivation and degree of staggering of the Cs-I planes as measured on the 3ML Cs-Pb-I-Cl nanoplatelets, to provide a realistic simulation. The stacking disorder parameter was instead changed linearly to account for the higher disorder expected for thicker nanoplatelets. As reference values we chose 3ML = 0.4 Å (as measured in this work on Cs-Pb-I-Cl) and 13ML = 1.4 Å (as measured in Ref. 2 on $CsPbBr_3$ nanocrystal superlattices).

S10. References

(1) Zhang, Y.; Siegler, T. D.; Thomas, C. J.; Abney, M. K.; Shah, T.; De Gorostiza, A.; Greene, R. M.; Korgel, B. A. A "Tips and Tricks" Practical Guide to the Synthesis of Metal Halide Perovskite Nanocrystals. *Chem. Mater.* **2020**, *32*, 5410–5423.

(2) Toso, S.; Baranov, D.; Altamura, D.; Scattarella, F.; Dahl, J.; Wang, X.; Marras, S.; Alivisatos, A. P.; Singer, A.; Giannini, C.; Manna, L. Multilayer Diffraction Reveals That Colloidal Superlattices Approach the Structural Perfection of Single Crystals. *ACS Nano* **2021**, *15*, 6243–6256.
